# Unified Workflow for the Rapid and In-Depth Characterization of Bacterial Proteomes

**DOI:** 10.1016/j.mcpro.2023.100612

**Published:** 2023-06-29

**Authors:** Miriam Abele, Etienne Doll, Florian P. Bayer, Chen Meng, Nina Lomp, Klaus Neuhaus, Siegfried Scherer, Bernhard Kuster, Christina Ludwig

**Affiliations:** 1 Bavarian Center for Biomolecular Mass Spectrometry (BayBioMS), TUM School of Life Sciences, Technical University of Munich, Freising, Germany; 2 Division of Proteomics and Bioanalytics, TUM School of Life Sciences, Technical University of Munich, Freising, Germany; 3 Division of Microbial Ecology, TUM School of Life Sciences, Technical University of Munich, Freising, Germany; 4 Core Facility Microbiome, ZIEL – Institute for Food & Health, TUM School of Life Sciences, Technical University of Munich, Freising, Germany

**Keywords:** bacterial proteomics, microflow, library-free data-independent acquisition, phylogenetic diversity, microbes

## Abstract

Bacteria are the most abundant and diverse organisms among the kingdoms of life. Due to this excessive variance, finding a unified, comprehensive, and safe workflow for quantitative bacterial proteomics is challenging. In this study, we have systematically evaluated and optimized sample preparation, mass spectrometric data acquisition, and data analysis strategies in bacterial proteomics. We investigated workflow performances on six representative species with highly different physiologic properties to mimic bacterial diversity. The best sample preparation strategy was a cell lysis protocol in 100% trifluoroacetic acid followed by an in-solution digest. Peptides were separated on a 30-min linear microflow liquid chromatography gradient and analyzed in data-independent acquisition mode. Data analysis was performed with DIA-NN using a predicted spectral library. Performance was evaluated according to the number of identified proteins, quantitative precision, throughput, costs, and biological safety. With this rapid workflow, over 40% of all encoded genes were detected per bacterial species. We demonstrated the general applicability of our workflow on a set of 23 taxonomically and physiologically diverse bacterial species. We could confidently identify over 45,000 proteins in the combined dataset, of which 30,000 have not been experimentally validated before. Our work thereby provides a valuable resource for the microbial scientific community. Finally, we grew *Escherichia coli* and *Bacillus cereus* in replicates under 12 different cultivation conditions to demonstrate the high-throughput suitability of the workflow. The proteomic workflow we present in this manuscript does not require any specialized equipment or commercial software and can be easily applied by other laboratories to support and accelerate the proteomic exploration of the bacterial kingdom.

## Highlights

• Bacteria are diverse organisms; therefore finding a unified proteomics workflow is challenging.
• Best performances were achieved with 100% TFA cell lysis and in solution digestion.
• Rapid and robust 30-min microflow LC in combination with DIA enabled high-throughput applications.
• Among 23 bacterial species, over 40% of all encoded genes were detected.
• We identified >30,000 proteins which have not been experimentally validated before.

## Graphical Abstract

**Figure:**
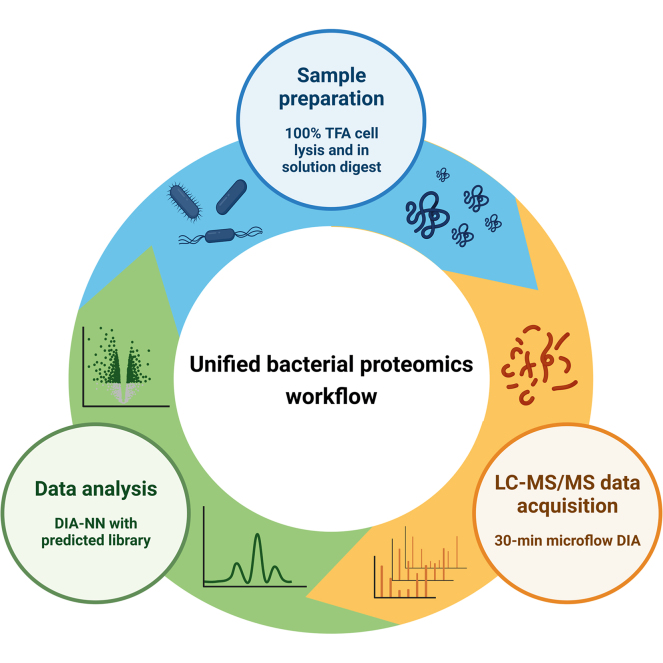
This Graphical abstract was created with Biorender.com.

## In Brief

Bacteria are highly diverse organisms, therefore, finding a unified proteomics workflow is challenging. We found that a 100% TFA cell lysis, in solution digest with a 30-min microflow liquid chromatography data-independent acquisition mass spectrometric analysis performed best regarding cost, safety, throughput, reproducibility, and proteome coverage. We applied this workflow to 23 diverse bacterial species and found experimental evidence for over 30,000 proteins.

Bottom-up proteomics has revolutionized microbiological research in many aspects. It paved the way for the qualitative and quantitative characterization of entire bacterial proteomes (1) and allowed the identification of undiscovered proteins (2, 3). Furthermore, the interaction of microbiological communities (4) provided new insights into their impact on human health (5), and quantitative proteomics shed light on the control of gene expression in *Escherichia coli* (6, 7). Many of these applications require a high sample throughput and profit from complete proteome coverages and accurate quantitative information. Therefore, a cost- and time-efficient, standardized sample preparation workflow for bacteria in combination with a short, single-shot LC-MS/MS method is desirable. However, most proteomic workflows have been optimized for eukaryotic sample types and applied to relatively few bacterial species (8, 9, 10, 11).

**Fig. 1:**
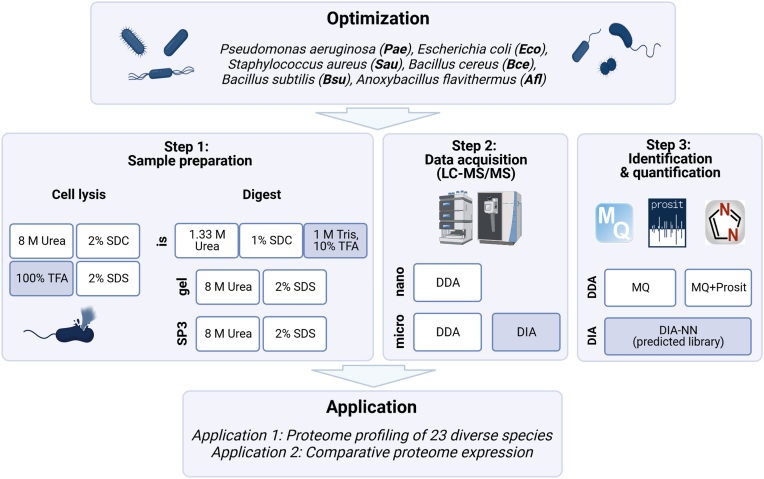
**Optimization of a proteomic workflow for protein expression profiling in bacteria.** Six diverse bacterial species (*top panel*) were subjected to four cell lysis strategies and compatible protein digestion protocols (*middle panel*; Step 1). Mass spectrometric data acquisition was performed in DIA and DDA mode on a Quadrupole-Orbitrap mass spectrometer equipped with a nano- or microflow liquid chromatography system (Step 2). DDA data were analyzed using MaxQuant with or without Prosit re-scoring. DIA data was analyzed using DIA-NN (Step 3). The approach that yielded the best result in each step is highlighted in dark blue. The optimized workflow was applied to protein expression profiling of 23 diverse bacterial species and a high-throughput example. The figure was created with BioRender.com. Drawing of the Ultimate3000 and Exploris 480 mass spectrometer, courtesy of Thermo Fisher Scientific. DDA, data-dependent acquisition; DIA, data-independent acquisition; gel, in-gel digest; is, in-solution digest; MQ, MaxQuant search with 1% protein false discovery rate (FDR) filtering; MQ+Prosit, MaxQuant search with 100% protein FDR followed by Prosit re-scoring and a picked-group FDR approach (1% FDR); SDC, sodium deoxycholate; SDS, sodium dodecyl sulfate; SP3, single-pot, solid-phase-enhanced sample-preparation; TFA, trifluoroacetic acid.

Every bottom-up proteomic experiment consists of at least three fundamental steps: (i) sample preparation, (ii) data acquisition, and (iii) protein identification and quantification (Fig. 1). Cell lysis is achieved with lysis buffers such as chaotropic agents (*e.g.*, urea or guanidinium hydrochloride) and detergents (*e.g.*, sodium dodecyl-sulfate or sodium deoxycholate). Lysis buffers should ideally efficiently extract and solubilize all proteins. Recently, 100% trifluoroacetic acid (TFA) was proposed as an alternative lysis strategy for proteomics (SPEED protocol, (12)). Since many bacteria are human pathogens, lysis buffers should completely inactivate vegetative cells and spores. Especially bacteria with a rigid cell wall (like *Mycobacterium* spp.) or bacteria that form resilient spores (like *Bacillus* spp.) can withstand even harsh lysis conditions. Applying mechanical forces like bead-beating (BB) or ultrasonication can support effective cell lysis (13, 14). Next, proteins are proteolytically digested with a sequence-specific protease like trypsin, LysC, or a combination thereof. Salts, chaotropes, and detergents must be removed before LC-MS/MS analysis because they reduce ionization efficiency and distort chromatography. Effective methods for this purpose are chaotropic dilution combined with solid phase extraction, protein precipitation, or gel electrophoresis.

Data are typically acquired on a liquid chromatography (LC) system coupled to a mass spectrometer. Prokaryotes have much smaller genomes and proteomes than eukaryotes. On average, bacteria encode for about 3750 genes (bacterial reference proteomes Uniprot (15)), while mammalian cells encode for 20,000 to 25,000 genes, and plant genomes are even larger (16). Consequently, the comprehensive coverage of bacterial proteomes may require less analytical effort. For example, shorter LC gradient times may support higher sample throughput. For many years, LC operated in nanoliter flowrates (nanoflow LC) dominated the field due to its high sensitivity. However, this often comes at the cost of robustness and the scale at which experiments can be conducted. Recently, microflow LC has become a viable alternative (17). It is highly reproducible, quantitatively precise, and more robust than nanoflow systems (17, 18, 19). The downside is a lower sensitivity owing to a lower electrospray ionization efficiency. This can be compensated by injecting higher peptide amounts for the analysis (18, 20).

Also, within a bacterial cell, the MS-detectable dynamic range of protein expression is smaller than in eukaryotic cells (around four orders of magnitude in a bacterial cell (21) *versus* at least seven orders in a human cell (22)). This favors a more comprehensive analysis of proteomes by MS/MS. To date, two data acquisition concepts are most frequently used. In data-dependent acquisition (DDA), the most intense peptide precursor ions are picked for fragmentation, and peptide sequences are determined from the resulting MS2 spectra by database searching using algorithms such as Mascot (23), MSFragger (24), or Andromeda (25). With the recent availability of spectrum prediction tools such as Prosit (26), database search results can be improved by better discriminating between true and false peptide-spectrum matches. In data-independent acquisition (DIA), (ideally) all peptides in a given sample are fragmented systematically through larger precursor isolation windows. This leads to more complex MS2 spectra that are analyzed by software tools such as Spectronaut (Biognosys AG) or DIA-NN (27). Traditionally, experimental spectra are compared to a library previously generated by DDA (28, 29). Alternatives are spectrum-centric approaches (30) or *in silico* predicted libraries (31), with which the drawback of laborious experimental library generation can be avoided. Critical to these so-called ‘library-free’ strategies are carefully controlled scoring algorithms and correct false-discovery rate estimations. Bacteria pose an easier statistical error control with their small proteomes, supporting genome-wide predicted spectral libraries.

Here, we present the systematic evaluation and optimization of seven sample preparation and data acquisition methods to develop a cost- and time-effective workflow that can be universally applied in bacterial proteomics. We selected six diverse, representative bacterial species for this evaluation. Our results demonstrate that bacteria can be inactivated entirely in 100% TFA. Applying a 30-min gradient on a microflow LC system, a DIA method, and a data analysis strategy based on an *in silico* predicted genome-wide spectral library, we could detect 40% of all open reading frames on average. We demonstrate the general applicability of this proteomic workflow by analyzing 23 taxonomically and physiologically diverse bacterial species. Finally, we analyze *E. coli* and *Bacillus cereus* grown under various cultivation conditions, thus demonstrating the high-throughput applicability of this workflow.

## Experimental Procedures

### Experimental Design and Statistical Rationale

We used three technical replicates to cover variation from cell lysis, sample preparation, and LC-MS/MS measurement for every combination of lysis and sample preparation strategy but excluded biological variance. Only one replicate was acquired for optimization experiments (Fig. 1; Step 1–4) and the 23 species experiment (Fig. 1; Application 1). *Bacillus cereus* and *E. coli* cultivated on different media types were carried out in triplicates, and samples were randomized before LC-MS/MS measurement. HeLa quality control samples have been measured before and after each bacterial batch to ensure equal LC-MS/MS performance. No spiked protein or peptide standards were used. For LC-MS/MS method optimization, technical replicate injections were carried out. MS1 data were acquired for all samples. DIA methods entailed different numbers of windows and widths to ensure a constant number of data points per peak (details in supplemental Table S1). All uniquely identified (quantified and non-quantified) peptides and proteins were reported. Proteins and peptides were filtered for multiple peptides per protein (“Sequence” in MaxQuant evidence.txt output file) for all experiments except for the TFA hydrolysis experiment. A Student’s *t* test was employed for differential protein abundance for the GO enrichment analysis and is described in the respective section. A Welch test was applied for differential expression analysis of bacteria grown under various cultivation conditions. A more detailed overview of all sample preparation optimization experiments (supplemental Table S2), cultivation conditions (supplemental Table S3), MS parameters (supplemental Tables S1 and S4), and analysis parameters (supplemental Table S5) are summarized in the supplemental Tables. A summary table of all acquired raw files can be found in the MassIVE repository.

### Cell Culture

All cultures were stored in glycerol stocks at −80 °C. To activate cultures, bacteria were passaged at least two times before starting an experiment. If not otherwise stated, bacteria were cultured on their respective agar plate types, temperatures, and aerobic conditions for 24 h (supplemental Table S3) and directly sampled from agar plates.

### Cell Lysis

In the case of sample preparation optimization experiments (Fig. 1, Step 1), samples were taken from agar plates, resuspended in TSB to an OD_600_ = 1, and washed once with phosphate-buffered saline (PBS). Per lysis and sample preparation combination (seven combinations, three replicates each), 2 ml of this suspension culture were centrifuged for 5 min at 10,000 rcf, and the supernatant was removed. For all other experiments, samples were taken from agar plates and directly lysed with 100% TFA.

Cell lysis was performed with four different lysis strategies, namely, Urea (8 M urea, 100 mM Ammonium bicarbonate, 5 mM ethylenediaminetetraacetic acid, 0.1 mM dithiothreitol (DTT)), sodium dodecyl sulfate (SDS, 2%), sodium deoxycholate (SDC, 2%), and trifluoroacetic acid (TFA, 100%).

#### SDS, SDC, and Urea

The cell pellet was suspended in 500 μl of the corresponding lysis buffer. In the case of SDS and SDC, samples were additionally heated for 10 min at 95 °C. An additional BB step was optionally included to test the influence of mechanical cell disruption on inactivation in the inactivation efficiency experiment. BB was performed with 0.1 mm silica beads in a FastPrep Tissue Homogenizer (MP Biomedicals, only half of the beads were used) by applying three cycles at 6.5 m/s speed for 45 s with a 90 s break between each cycle. Samples were centrifuged for 5 min at 10,000 rcf, and the supernatant was collected. Of note, only samples with BB were further processed for LC-MS/MS measurements.

#### TFA

100% TFA lysis was performed according to the SPEED protocol (12) with slight adaptations. Shortly, 50 μl of 100% TFA was added to the pellet, heated for 5 min at 55 °C, and then neutralized with 450 μl 2 M Tris (pH not adjusted). The pH after neutralization was around 8.2. Note that the TFA protocol was applied to samples from both applications (Fig. 1; Applications).

### In-Gel Protein Digest

In-gel protein digestion with trypsin was performed according to standard procedures (32). SDS + BB and Urea + BB samples were mixed with 4× NuPAGE Lithiumdodecylsulfat (LDS) sample buffer (ThermoFisher Scientific) to a final concentration of 1× LDS, and the SDS + BB samples were heated at 95 °C for 10 min. Thirty microliters were loaded on a NuPAGE 4% to 12% Bis-Tris Protein Gel (ThermoFisher Scientific). The gel was run for 4 to 5 min until proteins migrated around 1 cm into the gel (200 V, 500 mA, 1× 3-(N-morpholino)propane sulfonic acid buffer). Gels were fixated for 30 min (40% methanol, 2% acetic acid), colored with 1× RotiBlue (Carl Roth), and destained (25% ethanol (EtOH), 1% acetic acid). The bands were excised from the gel and transferred to a 96-well plate. Proteins were reduced (10 mM DTT in 5 mM triethylammonium bicarbonate (TEAB), 45 min, 55 °C), alkylated (55 mM chloroacetamide (CAA) in 5 mM TEAB, 30 min, room temperature), washed with 100 µl 5 mM TEAB, and dried with two times 100 µl EtOH. A volume of 20 μl (in the case of larger gel pieces, 40 μl) was added to the dried gel pieces (10 ng/μl trypsin in 5 mM TEAB, 15 min, 4 °C). Next, 20 μl (or 40 μl) 5 mM TEAB buffer was added, and proteins were digested overnight. Peptides were extracted by consecutively adding 5 μl of 5% formic acid (FA) (no incubation), two times 20 μl 1% FA (30 min, room temperature), 20 μl 60% acetonitrile (ACN)/0.1% FA (30 min, room temperature), 20 μl ACN (15 min, room temperature), and 20 μl ACN (15 min, room temperature). Between steps, samples were centrifuged for 1 min at 210 rcf. The generated peptides were dried in a vacuum concentrator and dissolved in 25 μl 0.1% formic acid (FA) in water.

### In-Solution Protein Digest

Protein concentrations were determined either by a Pierce BCA protein assay kit (Thermofisher; SDS + BB, SDC + BB, and Urea + BB samples) or by a Bradford assay (Thermofisher; TFA samples) according to manufacturer’s instructions. For digestion, 20 μg proteins were reduced and alkylated (TFA: 9 mM tris(2-carboxyethyl)phosphine (TCEP) and 40 mM CAA for 5 min at 95 °C, all others: 10 mM DTT for 30 min at 30 °C and 50 mM CAA for 30 min in the dark at room temperature). SDS + BB, SDC + BB, and Urea + BB samples were diluted with 50 mM ammonium bicarbonate to a final concentration of 1% SDS, 1% SDC, and 1.33 M urea. TFA samples were diluted with water to a final concentration of 1 M Tris and 5% TFA. Trypsin was added in a ratio of 1:50, for example, 0.4 μg for 20 μg protein input, to all samples and incubated overnight at 30 °C and 450 rpm (ThermoMixer C, Eppendorf). The next day, samples were acidified to a final concentration of 1% FA (in case of lysis with SDS, SDC, Urea) or 3% FA (in case of lysis with 100% TFA). SDC + BB samples were centrifuged at 845 rcf for 3 min, and the pellet was washed with 0.1% FA to remove precipitated SDC.

### SP3 Protein Digest

SDS + BB and Urea + BB samples were subjected to an SP3 deep well digest with an Agilent Bravo pipetting robot. The protocol was adapted from (33) and further edited. Briefly, all samples were diluted with the corresponding lysis buffer to a final volume of 100 μl and a protein concentration of 0.2 μg/μl. Magnetic beads (Sera-Mag A and B) were combined in an equimolar ratio and washed three times with deionized water. In the first step, samples were loaded into a 96-deep-well plate. A 1:7.5 protein to beads ratio was added to the samples. Proteins were precipitated by adding EtOH to a final concentration of 70%. Samples were washed thrice with 200 μl 80% EtOH and once with 100% ACN. Reduction and alkylation took place in 100 μl digestion buffer (100 mM 4-(2-hydroxyethyl)-1-piperazineethanesulfonic acid (HEPES) pH 8.5, 10 mM TCEP, 50 mM CAA) for 60 min at 37 °C and 1000 rpm (ThermoMixer C, Eppendorf). Trypsin was added in a 1:50 ratio. Samples were incubated overnight at 37 °C and 800 rpm (ThermoMixer C, Eppendorf). The following day, samples were acidified with TFA (1% final concentration), and peptides were desalted *via* C18 peptide cleanup.

### C18 Peptide Cleanup and Offline Basic pH Reversed-Phase Fractionation

All SP3 and in-solution digested samples were desalted before LC-MS/MS measurement. Self-packed desalting tips (34) were prepared in-house with three Empore C18 (3M) disks. Tips were primed with 100% ACN, then 40% ACN in 0.1% FA, and equilibrated with 0.1% FA. Peptides were loaded, washed with 0.1% FA, and eluted twice with 40% ACN in 0.1% FA. Fractionated samples were sequentially eluted with 5%, 7.5%, 10%, 12.5%, 15%, 17.5%, and 50% ACN in 25 mM ammonium formate resulting in eight fractions. Fraction 1 (5% ACN) and fraction 7 (50% ACN) as well as fraction 6 (17.5%) and the flow-through were pooled resulting in six fractions. Samples were lyophilized before storage at −80 °C.

### LC-MS/MS Measurements—Nanoflow Data-Dependent Acquisition

Two different nanoflow LC-MS/MS setups were employed in this study. For cell lysis, sample preparation optimization (Fig. 1; step 1) and data acquisition optimization (Fig. 1; step 2), a Dionex Ultimate 3000 RSLCnano system coupled to a Q-Exactive HF-X mass spectrometer (ThermoFisher Scientific) was used. For deep proteome profiling (fractionated samples) of the 23 diverse bacterial species (Fig. 1; Application 1), peptides were analyzed on a Dionex Ultimate 3000 RSLCnano system coupled to an Orbitrap Fusion Lumos Tribrid Mass Spectrometer (Thermofisher Scientific, Bremen). For single shot experiments during LC method optimization, 0.25 μg of peptides were delivered to a trap column (ReproSil-pur C18-AQ, 5 μm, Dr Maisch, 20 mm × 75 μm, self-packed) at a flow rate of 5 μl/min in HPLC grade water with 0.1% (v/v) FA. In the case of fractionated samples, 0.25 μg to 0.375 μg of peptides were injected. After 10 min of loading, peptides were transferred to an analytical column (3 μm C18 resin, ReproSil Gold Dr Maisch, 450 mm × 75 μm, self-packed) and separated using a 60 min linear gradient from 4% to 32% of solvent B (0.1% (v/v) FA, 5% (v/v) dimethylsulfoxide (DMSO) in ACN) at 300 nl/min flow rate. The nano-LC solvent A was 0.1% (v/v) FA and 5% (v/v) DMSO in HPLC-grade water.

The Q-Exactive HF-X (or Fusion Lumos in brackets if parameters differ) mass spectrometer was operated in data-dependent acquisition (DDA) and positive ionization mode. MS1 spectra (360–1300 m/z) were recorded at a resolution of 60,000 using an automatic gain control target value of 3 × 10^6^ (4 × 10^5^) and a maximum injection time of 45 ms (50 ms). Up to 18 peptide precursors (within a cycle time of 2 s) were selected for fragmentation. Only precursors with charge state two to six were selected, and dynamic exclusion of 25 s was enabled. Peptide fragmentation was performed using higher energy collision-induced dissociation and normalized collision energy of 26% (30%). The precursor isolation window width was set to 1.3 m/z (0.7 m/z). MS2 Resolution was 15,000 with an automatic gain control target value of 1 × 10^5^ and 25 ns maximum injection time. For the gradient optimization, the time of linear gradients was adjusted accordingly.

### LC-MS/MS Measurements—Microflow Data-Dependent Acquisition

For data acquisition optimization (Fig. 1, step 2), peptides were analyzed on a Vanquish Neo (microflow configuration) coupled to an Orbitrap Exploris 480 mass spectrometer (Thermo Fisher Scientific). Twenty micrograms of peptides were applied onto a commercially available Acclaim PepMap 100 C18 column (2 μm particle size, 1 mm ID × 150 mm, 100 Å pore size; Thermo Fisher Scientific) and separated using either a linear gradient (short gradients from 5 to 30 min) or a stepped gradient (for long gradients from 60 to 120 min; supplemental Table S1). Short gradients ranged from 3% to 28%, and long gradients from 3% to 31% of solvent B (0.1% FA, 3% DMSO in ACN) in solvent A (0.1% FA, 3% DMSO in HPLC grade water). A flow rate of 50 μl/min was applied. The mass spectrometer was operated in data-dependent acquisition (DDA) and positive ionization mode. MS1 full scans (360–1300 m/z) were acquired with a resolution of 60,000, a normalized automatic gain control target value of 100%, and a maximum injection time of 50 ms. Peptide precursor selection for fragmentation was carried out using various cycle times depending on the gradient length to keep a constant number of data points per peak (supplemental Table S1). Only precursors with charge states from two to six were selected, and dynamic exclusion of 35 s was enabled. Peptide fragmentation was performed using higher energy collision-induced dissociation and normalized collision energy of 28%. The precursor isolation window width of the quadrupole was set to 1.1 m/z. MS2 spectra were acquired with a resolution of 15,000, a fixed first mass of 100 m/z, a normalized automatic gain control target value of 100%, and a maximum injection time of 40 ms. For gradient optimization, the time of linear gradients was adjusted accordingly.

### LC-MS/MS Measurements—Microflow Data-Independent Acquisition

For data acquisition optimization (Fig. 1; Step 2), short gradient proteome profiling of 23 diverse bacterial species and the high-throughput experiment (Fig. 1; Applications) a Vanquish Neo (microflow configuration) coupled to an Orbitrap Exploris 480 mass spectrometer (Thermo Fisher Scientific) was employed. The LC gradient was identical to the micro-DDA method described above. The mass spectrometer was operated in data-independent acquisition (DIA) and positive ionization mode. MS1 full scans (360–1300 m/z) were acquired with a resolution of 120,000, a normalized automatic gain control target value of 100%, and a maximum injection time set to ‘Custom’. Peptide fragmentation was performed using higher energy collision-induced dissociation and normalized collision energy of 28%. Overlapping isolation windows were used (1 m/z, supplemental Table S4). MS2 spectra were acquired with a resolution of 15,000 (200–1800 m/z), a normalized automatic gain control target value of 100%, and a maximum injection time of 22 ms. For gradient optimization, the time of linear gradients was adjusted accordingly (supplemental Table S1).

### LC-MS/MS Data Analysis—MaxQuant

For all DDA measurements, MaxQuant (version 1.6.3.4) with its built-in search engine Andromeda (25, 35) was used for peptide identification and quantification. MS2 spectra were searched against all protein sequences obtained from UniProt (supplemental Table S5), supplemented with common contaminants (built-in option in MaxQuant). For *Anoxybacillus flavithermus*, we used a protein database previously published by Dettling *et al.* (36). Trypsin/P was specified as the proteolytic enzyme. Precursor tolerance was set to 4.5 ppm, and fragment ion tolerance to 20 ppm. The minimal peptide length was defined as seven amino acids, and the “match-between-run” function was disabled. For proteome analyses, carbamidomethylated cysteine was set as fixed modification and oxidation of methionine and N-terminal protein acetylation as variable modifications. iBAQ calculation was enabled in all experiments, and LFQ calculation was allowed only for cell lysis and sample preparation optimizations. For all sample preparation experiments, results were adjusted to a 1% false discovery rate (FDR) on peptide spectrum match and protein level employing a target-decoy approach using reversed protein sequences. In all other experiments, Prosit was employed to re-score the data (intensity: HCD, model from 2020; iRT: model from 2019) (26). Briefly, results from a 100% FDR MaxQuant search were re-scored by Prosit, and a “picked group FDR” estimation approach was applied to filter the data matrix for 1% FDR as described by The *et al.* (37). Proteins were filtered for “only identified by site,” “Potential Contaminants,” “Reversed,” and no protein groups were reported. In the proteinGroups.txt file, the “Peptides” column was filtered for > one peptide, and in the case of the evidence.txt file, only proteins with two ‘Sequence’ identifications were reported.

### LC-MS/MS Data Analysis—DIA-NN

DIA-NN (27) was employed for DIA data analysis. The main DIA-NN report “report.txt” was used for all calculations. Corresponding fasta files (supplemental Table S5) were digested with maximal two missed cleavages. Peptide length was restricted from 7 to 30 peptides, and the precursor m/z range was set from 360 to 1300. N-terminal methionine excision was enabled. Cysteine carbamidomethylation was selected as a fixed modification, and methionine oxidation and N-terminal acetylation as variable modifications. The maximum number of variable modifications was set to five. All other parameters were default settings, including the 1% precursor FDR and enabled match between runs (MBR). The main DIA-NN report “report.txt” was used for all calculations, and proteins were filtered to a Lib.PG.Q.Value <0.01 with at least two detectable peptides per protein. In the case of the DDA *versus* DIA comparison (Fig. 1; Step 2), we used the “first-pass-results.txt” report, in which MBR has not been applied, to guarantee a fair comparison between DDA (without MBR) and DIA (without MBR). In this case, we filtered for a Global.PG.Q.Value <0.01.

### Sample Preparation for the Comparative Proteome Profiling Application

Bacteria were grown on their respective media (supplemental Table S3) and harvested after 6 h, 24 h, or 48 h. Samples from agar plates were transferred into reaction tubes with an inoculated loop. In the case of tryptic soy broth, 1 ml from a 7 ml culture in a cultivation tube was transferred into a reaction tube and centrifuged at 6000 rcf for 5 min. Afterward, the liquid medium was removed. All samples were lysed with the 100% TFA protocol. Protein concentration was determined with the Bradford assay according to manufacturer’s instructions. Per sample, 20 μg protein was digested in a 96-well plate. During incubation, samples were covered with plastic foil. As described above, desalting was performed in stage tips with the following exceptions: All samples were desalted in a 96-well format with a 3D-printed stage-tip mount compatible with a centrifuge. In between, samples were centrifuged at 210 rcf for 3 to 5 min, depending on how quickly the solvent went through the tip. Centrifugation steps for sample loading and elution were carried out at 23 rcf for 10 min. Peptides were lyophilized and measured with the same parameters described above for micro-DIA.

### Cost Calculations

Only chemicals and consumables were considered. Solvents, buffers (except lysis buffers), and reusable consumables were neglected. Detailed cost estimation is listed in supplemental Table S7.

### Bioinformatic Analysis

If not otherwise stated, the MaxQuant evidence.txt file was used for analysis. Exceptions included (i) CV calculations, where LFQ intensities of MaxQuant’s proteinGroups.txt file were used, and (ii) iBAQ distribution plot, where iBAQ values from the proteinGroups.txt file were used.

#### Principal Component Analysis

Z-scored, median-centric normalized, log_2_ transformed intensities were used as input for the Python module scikit-learn (sklearn.decomposition.PCA).

#### Coefficient of Variation Calculations

**Fig. 2:**
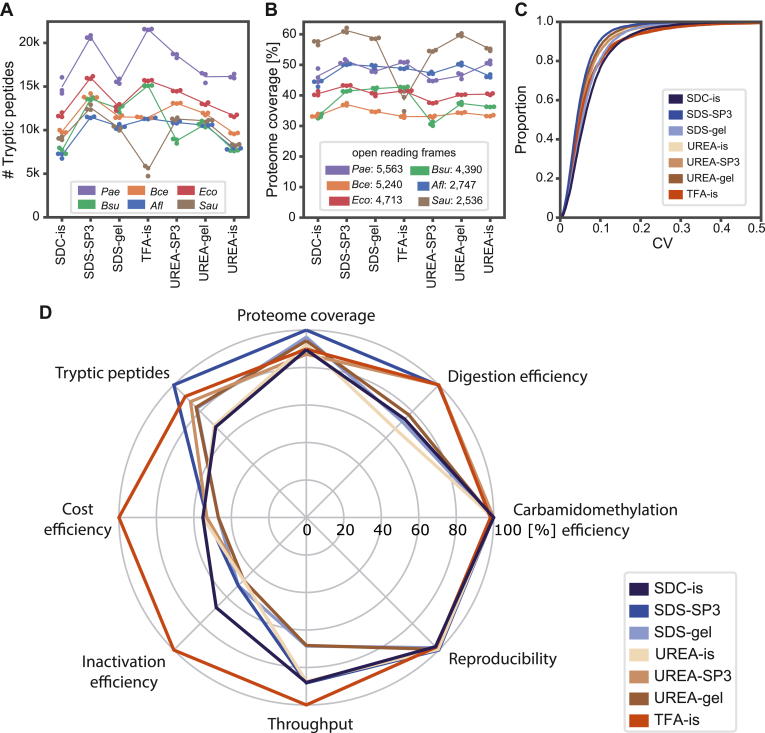
**Performance evaluation of seven sample preparation workflows.***A*, number of identified tryptic peptides (no missed cleavages) for the six bacterial species and seven sample preparations tested. *B*, same as (*A*) but for proteome coverage (percentage of identified proteins in relation to the number of open reading frame encoded genes). *C*, empirical cumulative distribution of proteins as a function of quantitative precision (coefficient of variation (CV) of proteins identified consistently in all protocols). CVs were calculated from technical triplicates. *D*, spider plot summarizing the performance of the sample preparation workflows according to the specified evaluation criteria. Parameters were normalized to the best-performing method in each category (refer to supplemental Table S10, tab 2D). gel = in-gel digest; is = in-solution digest; SDC = sodium deoxycholate; SDS = sodium dodecyl sulfate; SP3 = single-pot, solid-phase-enhanced sample-preparation; TFA = trifluoroacetic acid.

In the workflow optimization case, only proteins shared between all seven tested protocols were used (Fig. 2).

#### Gene Ontology (GO) Enrichment Analysis

*t* test was used to compare the mean protein expression between two sample preparation protocols. The significantly differentially expressed proteins were selected as *p*-value <0.05 and a fold change >2. Hits were subjected to an overrepresentation analysis using Fisher’s exact test. Sample preparation combinations with >4 significant GO terms (<0.05) were reported. Only GO terms that were detected in >2 comparisons were kept.

#### Volcano Plots

Differences in mean MaxLFQ intensities were calculated for each condition. *p*-values were computed with the Python package scipy.stats.ttest with equal_var =False (Welch test).

All other analyses were performed using in-house Python scripts with the help of the following Python packages: pandas, seaborn, matplotlib, numpy, sklearn, scipy (38), and matplotlib_venn.

## Results

### Comparative Evaluation of Bacterial Inactivation

To mimic the vast bacterial diversity existing in nature, six model species were selected for method evaluation and optimization: one Gram-positive, not spore-forming (*Staphylococcus aureus*, Sau), two Gram-positive, spore-forming (*Bacillus cereus*, *Bce*; *Bacillus subtilis*, *Bsu*), two Gram-negative (*Pseudomonas aeruginosa*, *Pae*; *E.* *coli*, *Eco*), and a thermophilic, Gram-positive, spore-forming bacterium (*A. flavithermus*, *Afl*). Since many bacteria are human pathogens and some are difficult to lyse, we evaluated the inactivation efficiency of four commonly used cellular lysis protocols: (i) 8 M Urea, (ii) 2% SDS, (iii) 2% SDC, and (iv) 100% TFA (12) (Fig. 1; Step 1). Colony-forming units per milliliter (cfu/ml) were determined for bacteria grown in culture before and after lysis. Inactivation efficiencies for all cell lysis strategies were, in general, high (>94.6%) but higher when cells were mechanically disrupted by BB (Table 1). For example, 140 and 723,000 cfu/ml were detected in 8 M Urea without BB for *Eco* and *Sau*, respectively. At the same time, an additional BB step resulted in no detectable cfu, corresponding to less than 10 cfu/ml. *Bsu* and *Afl* were the most resilient bacteria, with cfu detectable in most lysis strategies. Consequently, inactivation efficiencies dropped below 97%. The only protocol that inactivated all six bacterial species was the 100% TFA protocol. To back up this observation, we tested another 18 highly diverse bacterial species (supplemental Table S8). No colonies were observed for any bacterial species after treatment with 100% TFA. Since inactivation efficiencies were higher with an additional mechanical disruption step, we performed all following experiments with a BB step, except for the 100% TFA protocol, where BB was unnecessary.

**Table 1:** Bacterial inactivation efficiencies of 8 M Urea, 2% SDS, 2% SDC, and 100% TFA

Bacterium	Abbreviation	Lysis strategy	Cfu/ml before lysis	Detected cfu after lysis	Cfu/ml after lysis^a^	Inactivation efficiency [%]
*Pseudomonas aeruginosa* PAO1	*Pae*	8 M Urea	1.7 × 10^11^	0	<10	>99.9
		8 M Urea + BB		0	<10	>99.9
		2% SDS		0	<10	>99.9
		2% SDS + BB		0	<10	>99.9
		2% SDC		0	<10	>99.9
		2% SDC + BB		0	<10	>99.9
		100% TFA		0	<10	>99.9
*Escherichia coli* DSM 18039, K12 MG1655	*Eco*	8 M Urea	1.64 × 10^9^	1.40 × 10^1^	1.40 × 10^2^	>99.9
		8 M Urea + BB		0	<10	>99.9
		2% SDS		0	<10	>99.9
		2% SDS + BB		0	<10	>99.9
		2% SDC		0	<10	>99.9
		2% SDC + BB		0	<10	>99.9
		100% TFA		0	<10	>99.9
*Staphylococcus aureus* DSM 20231	*Sau*	8 M Urea	2.25 × 10^8^	7.23 × 10^4^	7.23 × 10^5^	>99.7
		8 M Urea + BB		0	<10	>99.9
		2% SDS		0	<10	>99.9
		2% SDS + BB		0	<10	>99.9
		2% SDC		0	<10	>99.9
		2% SDC + BB		0	<10	>99.9
		100% TFA		0	<10	>99.9
*Bacillus subtilis* DSM 10	*Bsu*	8 M Urea	1.94 × 10^8^	6.47 × 10^5^	6.47 × 10^6^	>96.7
		8 M Urea + BB		2.45 × 10^5^	2.45 × 10^6^	>98.7
		2% SDS		3.83 × 10^5^	3.83 × 10^6^	>98.0
		2% SDS + BB		9.45 × 10^4^	9.45 × 10^5^	>99.5
		2% SDC		5.27 × 10^3^	5.27 × 10^4^	>99.9
		2% SDC + BB		1.78 × 10^3^	1.78 × 10^4^	>99.9
		100% TFA		0	<10	>99.9
*Bacillus cereus* DSM 31	*Bce*	8 M Urea	9.09 × 10^7^	0	<10	>99.9
		8 M Urea + BB		0	<10	>99.9
		2% SDS		0	<10	>99.9
		2% SDS + BB		0	<10	>99.9
		2% SDC		0	<10	>99.9
		2% SDC + BB		0	<10	>99.9
		100% TFA		0	<10	>99.9
*Anoxybacillus flavithermus* WS5286	*Afl*	8 M Urea	6.95 × 10^5^	3.76 × 10^3^	3.76 × 10^4^	>94.6
		8 M Urea + BB		4.55 × 10^1^	4.55 × 10^2^	>99.9
		2% SDS		4.75 × 10^2^	4.75 × 10^3^	>99.3
		2% SDS + BB		4.85 × 10^2^	4.85 × 10^3^	>99.3
		2% SDC		6.75 × 10^1^	6.75 × 10^2^	>99.9
		2% SDC + BB		1.17 × 10^2^	1.17 × 10^3^	>99.8
		100% TFA		0	<10	>99.9

BB, bead-beating; Cfu, colony forming units.

a From undiluted lysate, only 100 μl were streaked on an agar plate. If no colony-forming units were detected after incubations, this translates to <10 colonies in 1 ml of lysate.

### Evaluation of Protein Hydrolysis by TFA

Since TFA is a strong organic acid, we tested if and to what extent acidic hydrolysis of proteins occurs. We hypothesized that the proportion of tryptic peptides decreases when hydrolysis occurs. Therefore, we incubated *Pae* and *Bce* in 100% TFA for 2 to 60 min and between 25 to 85 °C. Proteins were digested and analyzed by LC-MS/MS. Raw files were searched with MaxQuant allowing for tryptic and non-tryptic peptides (=‘unspecific’ search). One sample lysed with 8 M Urea and digested in solution was included as a control as no significant protein hydrolysis is expected under these conditions. With incubation times shorter than 10 min and temperatures of 55 °C, the 100% TFA protocol identified 16,800 peptides for *Pae* and 10,600 peptides for *Bce* (supplemental Fig. S1, *A* and *C*). Interestingly, for the spore-forming bacterium *Bce*, incubation at 55 °C led to more peptide identifications than at 25 °C. This might be attributed to better lysis of the resilient spores. The proportion of tryptic peptides was similar to those of Urea lysed samples (>94%) in all conditions with an incubation temperature of 25 °C and short incubation times (below 40 min) at 55°. In comparison, incubation for longer than 2 min at 85 °C led to a substantial time-dependent reduction of tryptic peptide identifications (supplemental Fig. S1, *B* and *C*), indicating protein hydrolysis at higher temperatures. Considering these observations, we used for all further experiments with the 100% TFA protocol an incubation time of 5 min at 55 °C to achieve high peptide identifications, especially for spore-forming bacteria and concomitantly avoiding hydrolysis.

### Comparative Evaluation of Standard Sample Preparation Methods

We compared the performance of three protein digestion methods: (i) in-solution (is), (ii) in-gel (gel), and (iii) single-pot, solid phase, enhanced sample preparation (SP3). Since not every lysis buffer is compatible with the above digestion methods, the following seven combinations were evaluated: TFA-is, Urea-is, Urea-SP3, Urea-gel, SDS-SP3, SDS-gel, and SDC-is (Fig. 1; Step 1). For all six bacteria, all sample preparation protocols were performed in technical triplicates, and at least two unique peptide sequences per protein were required for confident protein detection. Total protein yields were determined with a defined cell number corresponding to 2 ml of OD_600_ = 1 using either BCA or Bradford assay, depending on the assay’s compatibility with salts and detergents. All seven lysis protocols yielded broadly similar quantities for a particular species. However, protein yields varied between bacteria by up to two-fold, with *Sau* providing the lowest and *Eco* the highest (supplemental Fig. S2).

LC-MS/MS analysis led to noteworthy differences in the number of fully tryptic peptide identifications between the species, ranging from 4729 to 21,598. On average, SDS-SP3 and TFA-is were the two protocols that generated the highest number of detectable peptides (over all six bacterial species, Fig. 2*A*). Strikingly, around 18,560 peptides were detected for all *Sau* sample preparation protocols, except for the TFA-is protocol, which resulted in 6649 peptides and 977 proteins, only (Fig. 2, *A* and *B*). Next, we investigated the occurrence of missed-cleaved tryptic peptides. SDS-gel, Urea-gel, SDC-is, and Urea-is showed missed-cleavage rates of up to 51%, while SDS-SP3, Urea-SP3, and TFA-is showed missed-cleavage rates of around 27% (supplemental Fig. S3*A*). Alkylation efficiencies of cysteine residues were similarly high for all protocols (95.4% – 99.8%, supplemental Fig. S3*B*). Even the TFA-is protocol, where reduction and carbamidomethylation incubations were reduced from 1 h to 5 min, still showed acceptable efficiencies. The achieved proteome coverages, which we calculated as the percentage of identified proteins compared to all encoded genes, ranged from 34% (*Bce*) to 55% (*Sau*) (Fig. 2*B*). The overlap of all identified proteins between the seven tested protocols was 62.6%. Only 721 of the 13,629 identified proteins (5.2%) from all six species were exclusively found in one of the seven sample preparation approaches (supplemental Fig. S3*C*). The overlap between related protocols was even higher, with 73.9% shared proteins between the three in-solution digestion protocols and 77.6% shared proteins between the Urea-lysed protocols (supplemental Fig. S3, *D* and *E*).

We next determined the differentially abundant proteins between all seven protocols to identify quantitative biases. We performed an over-representative analysis (ORA) based on gene ontology (GO) terms (39, 40) independently for two well-annotated representative species, *Eco* and *Bce*. Interestingly, mainly membrane-related GO terms were enriched in samples digested in the gel. The TFA-is protocol enriched for ribosomal proteins, transcription, and translation-related GO-terms compared to Urea-lysed samples (supplemental Fig. S4, *A* and *B*) but showed no biases in contrast to other protocols. Quantitatively, all protocols showed a high precision with median coefficients of variance (CV) on protein level between 5.1% (SDS-SP3) and 7.6% (SDC-is) across all species (Fig. 2*C*).

Finally, we investigated the parameters “total costs” and “sample throughput” for each of the seven protocols (supplemental Tables S6 and S7). The TFA-is protocol requires the least hands-on time and the shortest incubation times for reduction and alkylation, thus supporting the highest sample throughput. This protocol is also cost-efficient as there are no costs for BB, magnetic beads for SP3 digest, or SDS gels. The two in-gel digestion protocols are substantially more expensive (around two times the TFA-is costs) and, in our hands, require about 100 h preparation time per 96 samples. Samples prepared with the SP3 workflow on a pipetting robot were the most expensive (around 1.8 times the TFA-is costs) with medium-time investments (∼19 h/96 samples).

Considering all performance characteristics investigated in this study (Fig. 2*D*), the TFA-is protocol was the best overall choice. It achieved similarly high numbers of peptide and protein identifications as other protocols and excellent reproducibility, but it excelled in terms of inactivation efficiency, sample throughput, and costs. Hence, we used the TFA-is protocol for all further experiments described below.

### Optimization of Single-Shot Microflow Gradient for Bacterial Proteomics

It has been shown that single-shot data-dependent acquisition by microflow LC-MS/MS identifies up to 9000 proteins within 3 h of measurement time in eukaryotic cellular lysates (19). As noted above, bacteria have substantially smaller proteomes, and protein expression spans a smaller dynamic range. Hence, we reasoned that (i) LC gradients may be shortened without diminishing proteome coverage and (ii) microflow LC (50 μl/min) may provide an alternative to nanoflow LC (300 nl/min) for global proteome profiling in bacteria.

**Fig. 3:**
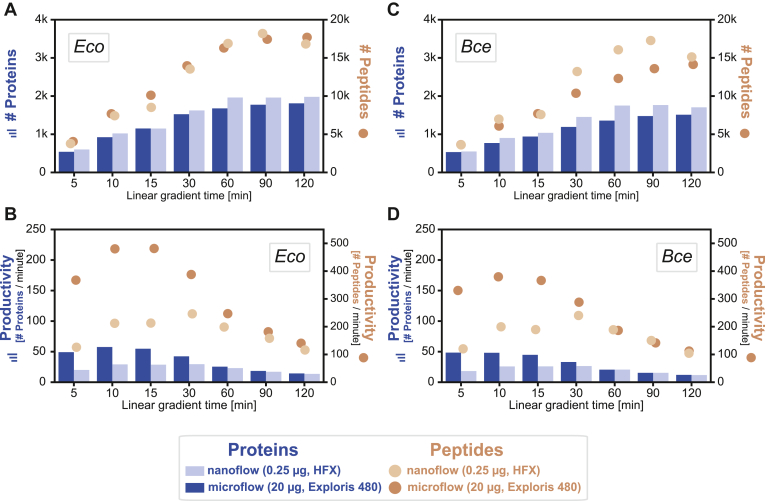
**Performance evaluation of micro- and nanoflow liquid chromatography.***A*, number of proteins (*bars*, *left axis*) and peptides (*circles*, *right axis*) identified for *Escherichia coli* as a function of linear gradient time using nanoflow (*light color*) and microflow (*dark color*). *B*, productivity of protein and peptide identification per minute as a function of total method time (linear gradient time plus overhead time). The x-axis label denotes the linear gradient time only. *C* and *D*, same as panels (*A* and *B*) but for *Bacillus cereus*.

To investigate the influence of gradient length and flow rate on the analysis of bacterial proteomes, we tested linear gradient lengths between 5 and 120 min with a microflow LC Orbitrap Exploris and a nanoflow LC Orbitrap HFX setup (Fig. 3). As expected, protein and peptide identifications increased with longer LC gradients but plateaued at around 60 min (1850 and 1600 protein identifications for *Eco* and *Bce*, respectively; Fig. 3, *A* and *C* and supplemental Fig. S5). We note that we injected 80 times more peptide quantity onto the microflow (20 μg) than onto the nanoflow systems (0.25 μg) to compensate for the reduced ionization efficiency at higher flow rates (20). Evaluating the dependency between peptide injection amount and gradient length for the microflow LC system showed no discernible differences between 5 and 20 μg for LC gradients of up to 60 min (supplemental Fig. S6). Longer gradients, however, require higher injection amounts of at least 10 μg for optimal performance. The flow rate of microflow LC separations comes with the benefit of shorter overhead times. Consequently, the productivity, defined as the number of protein or peptide identifications per minute of total method time (linear time plus overhead time), was higher on the microflow LC-MS/MS setup (Fig. 3, *B* and *D*). It peaked at a gradient length of around 15 min, resulting in about 400 peptide and 50 protein identifications per minute.

### Evaluation of DDA and DIA Methods for Bacterial Proteome Profiling

Next, we combined microflow LC separations with two different mass spectrometric data acquisition types, DDA, and DIA. We injected 10 μg of peptides. For the data analysis of the raw files generated in DDA mode, we used MaxQuant (MQ)/Andromeda (25) (DDA MQ) with and without Prosit re-scoring (MQ+Prosit) (26, 37) (details in the Experimental Procedures section). The software tool DIA-NN (27), with its built-in option for *in-silico* library prediction (“library-free”), was used to analyze the DIA data.

**Fig. 4:**
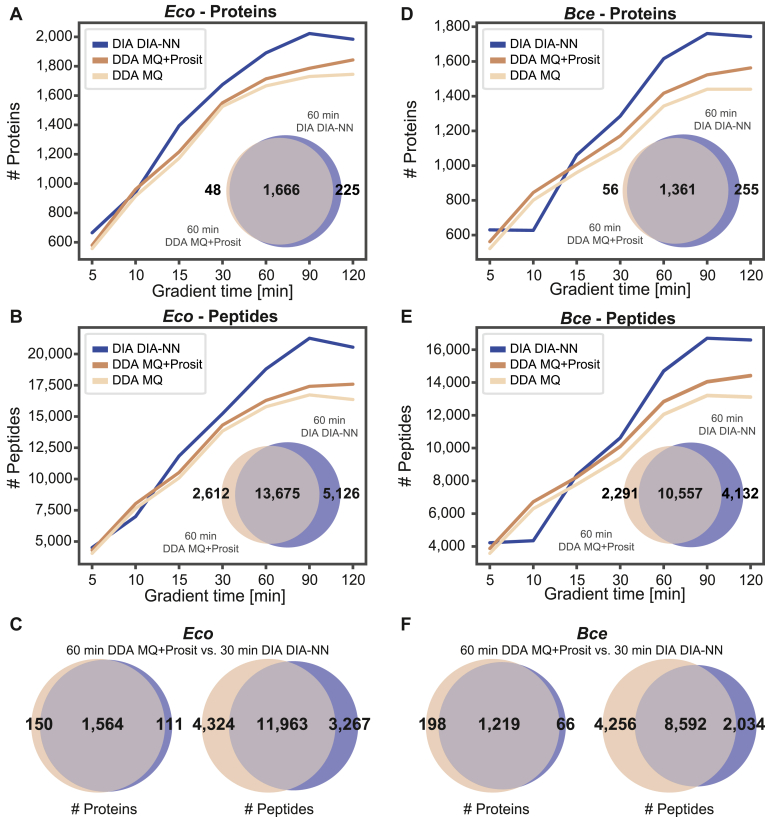
**Performance evaluation of data-dependent and data-independent acquisition.***A*, number of protein identifications for *Escherichia coli*. Ten micrograms of peptides were injected per run. DIA data were searched with an *in-silico* predicted library with DIA-NN (DIA DIA-NN, *dark blue line*). DDA data were analyzed in two ways: First, with MaxQuant using a 1% protein-level FDR (DDA MQ, *brown line*), and second, with a 100% FDR MaxQuant search followed by a Prosit re-scoring step and a picked group FDR estimation approach (DDA MQ+Prosit, beige line). Venn diagrams represent the protein overlap in a 60-min DIA DIA-NN experiment *versus* a 60-min DDA MQ+Prosit experiment. *B*, same as (*A*), but for peptide identifications. *C*, Venn diagrams show the overlap between a 60-min DDA MQ+Prosit experiment and a 30-min DIA DIA-NN experiment. *D*–*F*, same as panels (*A*–*C*) but for *Bacillus cereus*.

For all data acquisition and analysis types, protein identifications increased continuously with gradient length and eventually reached a plateau at 60-min gradients (Fig. 4). In general, MQ+Prosit resulted in higher numbers of peptide and protein identifications than non-re-scored DDA data for all analyzed gradients (Fig. 4, *A*, *B*, *D* and *E* and supplemental Fig. S7). For example, net gains of around 6% were obtained for samples acquired with a 60-min gradient (gain 10%, loss 4%; supplemental Fig. S8). DIA DIA-NN performed qualitatively better than DDA MQ+Prosit for LC gradients of 15 min and longer (Fig. 4, *A*, *B*, *D* and *E* and supplemental Fig. S7). For example, with a 60-min gradient, 1891 proteins were identified with DIA DIA-NN, and 1714 were identified with DDA MQ+Prosit for the model organism *Eco*. As expected, the protein overlap was very high (97%) between the two acquisition and analysis strategies (Fig. 4*A*). We compared a 30-min DIA DIA-NN method to a 60-min DDA MQ+Prosit method to achieve a higher sample throughput. We found that they resulted in almost identical protein numbers (Fig. 4, *C* and *F* and supplemental Fig. S7), thus covering the same proteome depth in bacteria.

### Proteome Expression Maps of 23 Highly Diverse Bacterial Species

**Fig. 5:**
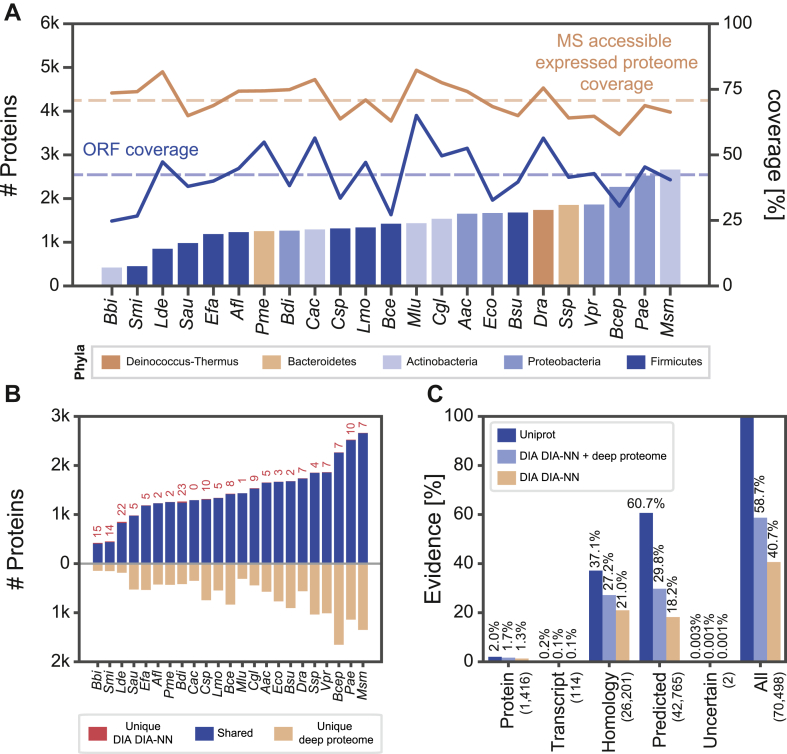
**Proteome profiling of 23 diverse bacterial species.***A*, number of detected proteins per species in a single shot 30-min DIA DIA-NN experiment (*bars*, *left axis*). *Bar colors* indicate the taxonomic relationship. Line plots show the open reading frame (ORF) coverage (*blue*, *right axis*) and MS accessible expressed proteome coverage (*brown*, determined by deep proteome profiling where proteomes were measured in six fractions, right axis). *B*, bar-type Venn diagrams comparing a 30-min DIA DIA-NN experiment to a deep proteome profiling experiment. Proteins uniquely identified in the 30-min DIA DIA-NN experiment are highlighted in *red*, shared proteins in *blue*, and proteins uniquely identified by deep proteome profiling in *beige*. *C*, evidence for the existence of a protein according to Uniprot’s annotations for each ORF (*dark blue*). Protein evidence from the deep proteome profiling plus DIA DIA-NN experiment (*light blue*) and the DIA DIA-NN profiling (*beige*). *Anoxybacillus flavithermus* was excluded from the analysis due to missing annotations in UniProt. *Mycolicibacterium smegmatis* and *Listeria monocytogenes* were excluded due to missing strain-specific fasta files (Uniprot).

From the results above, we concluded that a workflow combining TFA-is cell lysis and protein digestion with a 30-min microflow LC separation and DIA-MS/MS provided the best results for the six bacterial model species. Additionally, the *in-silico* predicted library option of DIA-NN enabled us to analyze all bacteria without the need for experimental library generation. To test whether this approach is more widely applicable, we expanded the list of bacteria by another 17 highly diverse species and analyzed their global proteomes. The choice of bacteria followed the selection criteria: (i) phylogenetic relationship, (ii) academic relevance (model organism), (iii) technological relevance, (iv) pathogenicity, and (v) lysis resistance (supplemental Table S9). With our optimized workflow, most proteins (2664) were found for *Mycobacterium smegmatis* (*Msm*), a model for the clinically relevant *Mycobacterium tuberculosis* (Fig. 5*A*). In contrast, *Bifidobacterium bifidum* (*Bbi*), with 422 protein groups, was the bacterium with the least proteins identified. We could not detect any identification bias towards one taxonomic class or family.

On average, we identified 42% of all open reading frames per species in 30 min of DIA measurement time (Fig. 5*A*, blue line). We further fractionated each bacterial sample using high-pH reverse phase fractionation to explore how many proteins were expressed by each bacterial species at the time of cell lysis. We used a 60-min DDA LC-MS/MS to analyze each fraction. The resulting ‘MS accessible expressed proteomes’ were compared to the single shot 30-min DIA measurements. Overall, we could detect, on average, 71% of the MS-accessible expressed proteomes with our 30-min DIA workflow (Fig. 5*A*, brown line). Interestingly, *Bbi*, the species with the least proteins identified, still had a very high MS-accessible expressed proteomes coverage of 74%. The vast majority of proteins (>99% on average) identified in the short DIA runs were confidentially identified in the fractionated DDA samples (Fig. 5*B*), and intensities of both runs correlated well (R^2^ > 0.76, supplemental Fig. S9). As expected, proteins exclusively identified in MS-accessible expressed proteomes generally have lower intensities (supplemental Fig. S9).

Uniprot’s protein evidence score provides information about the evidence of the existence of any given protein (15). The vast majority of bacterial proteins detected in this study were as “proteins inferred from homology” (36.7%) or as “proteins predicted” (61.1%) (Fig. 5*C*, *Afl* was excluded due to missing annotations on UniProt, *Msm*, and *Lmo* were excluded due to fasta files not matching the exact strain). With our high-quality single-shot DIA dataset, we increased the number of experimentally validated proteins from 1416 to 28,668 proteins, corresponding to 41% of all open reading frames from the 20 bacterial species investigated where annotations and strain-specific fasta files were available (Fig. 5*C*). The combined dataset, consisting of single-shot DIA and fractionated samples, provides proteomic evidence for 41,398 bacterial proteins, equivalent to 59% of all open reading frames. Our data increased the number of experimentally validated proteins for the 20 bacterial species described in this study by 30-fold (Fig. 5*C*).

### Time-Dependent Proteome Expression Analysis of Bacterial Growth on Various Media

**Fig. 6:**
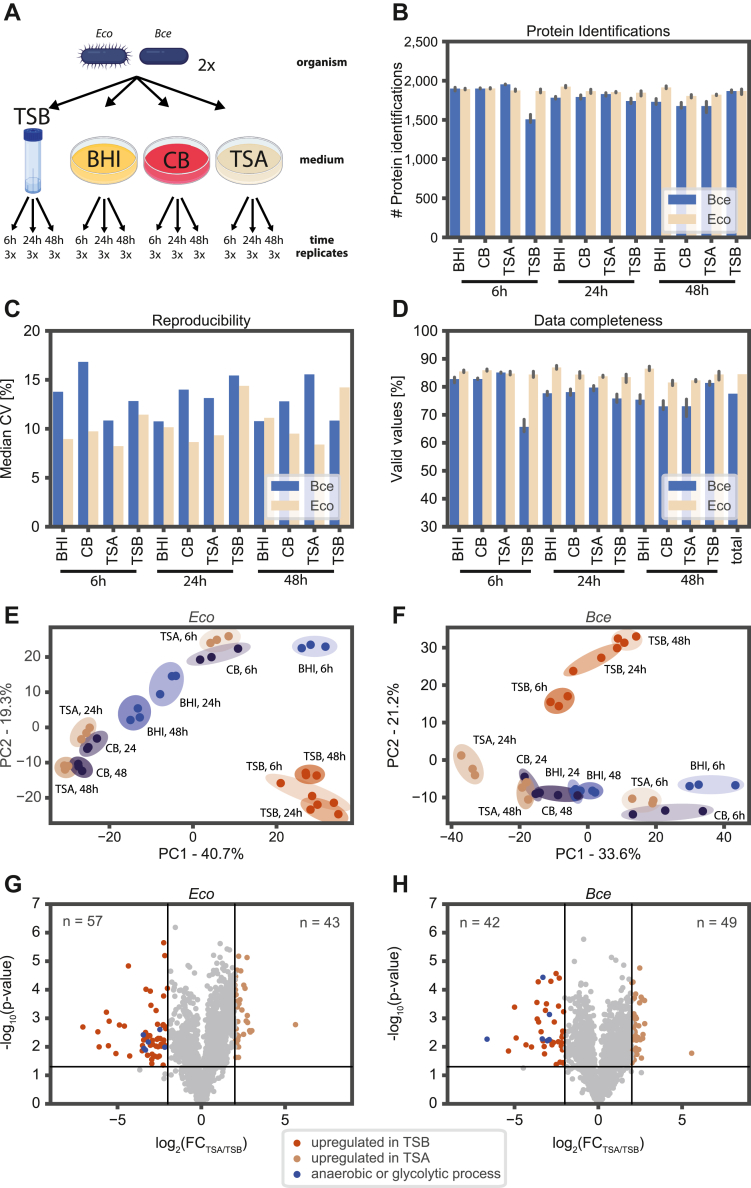
**Comparative proteome analysis of *Escherichia coli* and *Bacillus cereus* grown under diverse cultivation conditions.***A*, two organisms, *Escherichia coli* (*Eco*) and *Bacillus cereus* (*Bce*), were cultivated on four different media: Tryptic soy broth (TSB), Brain-Heart infusion (BHI), Columbia blood (CB), and Tryptic soy agar (TSA). Bacteria were harvested after 6 h, 24 h, and 48 h incubation time. All experiments were conducted in biological triplicates. *B*, number of protein identifications of *Escherichia coli* (*Eco*) and *Bacillus cereus* (*Bce*) for all samples. Error bars indicate a confidence interval of 0.95. *C*, reproducibility of the workflow as determined by the coefficient of variation (CV) from three biological replicates. *D*, data completeness is the ratio between the number of protein identifications of one sample and the union of protein identifications in the entire organism-specific dataset. Data completeness of the whole sample: protein matrix is reported under the term ‘Total’. Error bars indicate a confidence interval of 0.95. *E*, principal component analysis (PCA) for *Escherichia coli* based on log2-transformed MaxLFQ normalized intensities from the DIA-NN output file. *F*, same as (*E*), but for *Bacillus cereus*. *G*, volcano plot showing differentially expressed proteins of *Escherichia coli* cultivated for 24 h on either TSA or TSB. The ratio was calculated for each protein as the average MaxLFQ intensity of the three biological replicates. The *p*-value was calculated with a Welch test. *Red lines* represent significance cutoffs (*p*-value < 0.05, −4 > fold change > 4). Proteins highlighted in *blue* are involved in anaerobic or glycolytic processes. *H*, same as (*G*), but for *Bacillus cereus*. *Panel A* was created with BioRender.com and further modified.

We applied our rapid bacterial proteomics workflow to analyze bacterial proteome adaptations upon different cultivation conditions. In total, we analyzed 72 different samples derived from two bacterial strains (*E. coli* (*Eco*) and *Bacillus cereus* (*Bce*)) grown in biological triplicates on four media (Tryptic soy broth TSB; Brain-Heart infusion BHI; Columbia blood (CB); Tryptic soy agar TSA) for 6 h, 24 h, and 48 h (Fig. 6*A*). We processed all samples with the 100% TFA protocol in a 96-well format and measured the bacterial proteomes with the proposed 30-min microflow DIA method. Data analysis was performed with DIA-NN and predicted spectral libraries. Sample preparation of all 72 samples was completed in 25 h with only 6 h of hands-on time. Data acquisition took 44 h of measurement time (36 h linear gradient time + 8 h overhead time), and data analysis finished in 4.5 h. On average, we quantified 1870 proteins and 1780 proteins for *Eco* and *Bce*, respectively (Fig. 6*B*). The coefficient of variation over the three biological replicates ranged between 8% and 17% on protein level (Fig. 6*C*). In the two datasets, we achieved, on average, 84.6% (*Eco*) and 77.6% (*Bce*) data completeness (Fig. 6*D*). Samples from both bacterial strains showed distinct protein clusters in principal component analysis. Most prominently, cells grown in the liquid culture condition TSB clustered apart from all other samples in which bacteria were grown on agar (Fig. 6, *E* and *F*). We performed a differential protein abundance analysis between TSB and TSA to characterize proteomic adaptation processes. This analysis showed that *Eco* and *Bce* proteins involved in anaerobic respiration and glycolysis were downregulated in TSA compared to TSB (Fig. 6, *G* and *H*). This observation indicates that bacteria grown under mild agitation in cultivation tubes containing TSB switch their metabolism to fermentation due to limited oxygen supply.

The above-presented data illustrates the excellent suitability of the present workflow for high-throughput applications, in which 72 samples can be prepared, measured, and analyzed within 3.5 days, ultimately contributing to a better understanding of bacterial adaptations on a molecular level.

## Discussion

The present study provides an in-depth evaluation of seven widely used proteomics workflows for sample preparation in the context of bacterial proteomics. We investigated six highly diverse bacterial species and based our performance evaluation on various criteria reflecting the enormous biological diversity within the bacterial kingdom. Our results demonstrate that all tested protocols delivered a similar performance regarding detectable proteins/peptides and quantitative precision but showed significant differences in costs, biological safety, and throughput. In our hands, the 100% TFA in-solution protocol (12) outperformed all other protocols in the categories of inactivation efficiency, cost-effectiveness, and throughput. At the same time, it maintained excellent protein and peptide identification numbers and exact quantification results. Performing bacterial cell lysis with 100% TFA is easy and fast and does not require any BB step or specialized instrumentation. Interestingly, the total protein yield of samples lysed with 100% TFA was roughly similar to all tested protocols, which contradicts findings of other studies that report protein yields to increase by 54% with 100% TFA (12) and 1.8-fold in the case of detergents (8), both in comparison to Urea lysed samples. We further show that 100% TFA is the only protocol that efficiently inactivates 23 diverse bacterial species, including bacteria that form spores, which are notoriously hard-to-inactivate bacteria (13, 41). Although we did not observe any colony-forming units after 100% TFA treatment, bacterial samples always require extra checks for complete inactivation. TFA is a strong organic acid that can solubilize proteins but potentially hydrolyze peptide bonds (42). This unwanted side reaction would reduce the number of detectable tryptic peptides by bottom-up proteomics and falsify the quantitative proteomic results. The authors of the original SPEED protocol described in their publication that no protein hydrolysis effect from TFA incubation at room temperature for up to 60 min on the *E.* *coli* proteome (12). We confirmed this observation in our experiments, but we show that acid-induced protein hydrolysis occurs at higher temperatures and longer incubation times (>55 °C, >20 min). Additionally, we found that lysis of the spore-forming bacterium *Bacillus cereus* with 100% TFA is inefficient at room temperature, but higher temperatures (ideally 55 °C) are required. Besides all the positive effects of 100% TFA, it exhibited one weakness. *S.* *aureus* showed a significant drop in identifications with the 100% TFA-is sample preparation workflow compared to all others. This observation demonstrates that a universal workflow for bacterial proteomics with excellent performances in all categories for all bacterial species is hard to find if such one exists (11).

Since bacteria have smaller proteomes and a lower dynamic range than eukaryotes, we optimized an LC-MS/MS method for bacteria. A 30-min microflow LC DIA MS/MS method with *in-silico* predicted libraries provided the best compromise between full proteome coverages and throughput. Microflow LC has several advantages over nanoflow applications. It is (i) more reproducible, (ii) more robust, (iii) has higher peak-capacities, (iv) shorter overhead times, and (v) less sample carryover between adjacently measured samples (18). Protein identifications are comparable between microflow and nanoflow LC-MS/MS. For high-throughput applications, it is, therefore, the higher productivity due to shorter overhead times and its excellent robustness that renders microflow LC-MS/MS an attractive alternative to nanoflow LC-MS/MS. A drawback of microflow LC is its reduced ionization efficiency. However, we show that 2 ml of a suspension culture (OD_600_ = 1) yielded, on average, 0.7 mg of protein, which is enough starting material for a microflow LC-MS/MS run which requires between 0.02 and 0.04 mg protein (0.01–0.02 mg on peptide level). DIA is well suited for large-scale studies due to its high quantitative consistency and accuracy. It is not limited to the number of acquired MS2 spectra, thus supporting the use of short gradients (29). However, high peak capacities in microflow LC setups, in combination with a short gradient length, result in sharp peaks with a small average peak width. If one wants to keep the number of measured data points per peak sufficient (in the range of six to eight data points per peak), a trade-off can be that the DIA data needs to be acquired with relatively wide precursor isolation windows. This, in turn, leads to highly complex MS2 spectra, which negatively affects peptide identifications and accurate quantification. *In-silico* predicted libraries of bacteria require no empirical corrections (43) because their small genome size has a lower multiple testing burden than the substantially bigger eukaryotic proteomes. Especially in large-scale studies, computational times must be considered in the experimental design because they can become a bottleneck in the workflow. *In-silico* predicted libraries are typically extensive and thus require more computational resources than experimental libraries. However, we experienced that DIA-NN copes well with predicted prokaryotic libraries. For example, the 72 raw files from the high-throughput application were processed in around 4.5 h, thus being ten times faster than the mass-spectrometric acquisition time.

We applied the workflow advocated in this study to a set of 23 highly diverse bacterial species to demonstrate its general applicability. We achieved open reading frame (ORF) coverages as high as 65% (*Micrococcus luteus*) and MS-accessible expressed proteome coverages as high as 82% (*Lactobacillus delbrueckii*, *M. luteus*). On average, 40% of all ORFs and 70% of the MS-accessible expressed proteomes could be analyzed in a rapid, single-shot experiment, demonstrating the potential of this method for large-scale studies. With our high-quality dataset, we increased Uniprot’s protein evidence by 30-fold to almost 60% of all described ORFs for the 20 species described in this study (excluding *A. flavithermus*, *Mycolicibacterium smegmatis*, and *Listeria monocytogenes*). Although *E. coli* is a well-studied model organism where extensive proteomic investigations have been undertaken (21), we increased the number of newly described proteins from 48 to 2395. This is because previous studies were conducted mainly on laboratory strains, *e.g.*, NCM3722, MG1655, and BW25113. For example, in *E. coli* MG1655, 3079 from 4448 ORFs have been described on a proteomic level. The type-strain DSM 30083 we used in this study had only 48 of its 5098 ORFs described.

Finally, we used the proposed workflow for a comparative proteome expression study, consisting of 72 samples, to exemplify the advantages of this workflow for high-throughput applications. All samples were rapidly lysed with the 100% TFA protocol. The in-solution sample preparation workflow is 96-well format compatible. It can be performed either on a pipetting robot or, as in our case, manually (more details in the experimental procedure section). We described proteomic differences of bacteria grown under diverse cultivation conditions. For example, bacteria grown in liquid culture had higher expression levels of proteins involved in glycolysis and anaerobic metabolism. This observation points towards a lack of oxygen supply under mild agitation. At least part of the population switched their metabolism to anaerobic respiration. Higher agitation rates and/or baffled flasks can increase oxygen supply and thereby avoid the occurrence of bacterial cultures in mixed metabolic states. This example illustrates that experimental setup drastically influences the molecular state of bacteria, ultimately impacting the interpretation of proteomic results.

In conclusion, the optimized 100% TFA 30-min DIA workflow represents a safe, rapid, cost-effective, in-depth, and highly reproducible sample preparation workflow that opens up the potential for large-scale bacterial proteomic studies.

## Data Availability

The mass spectrometry proteomics data have been deposited to the ProteomeXchange Consortium (https://massive.ucsd.edu/ProteoSAFe/static/massive.jsp) *via* the MassIVE partner repository with the dataset identifier MSV000091088.

## Supplemental Data

This article contains supplemental data (36).

## Conflict of interest

The authors declare the following financial interests/personal relationships which may be considered as potential competing interests: Bernhard Kuster is co-founder and shareholder of OmicScouts and MSAID. He has no operational role in either company.
