# Supplementary material for: Unified Workflow for the Rapid and In-Depth Characterization of Bacterial Proteomes
Source: Mol Cell Proteomics. 2023 Jun 29;22(8):100612. doi: 10.1016/j.mcpro.2023.100612 (PMC10407251; doi:10.1016/j.mcpro.2023.100612)
Supplement: Supplemental Figures [file mmc1.docx]

**Unified workflow for the rapid and in-depth characterization of bacterial proteomes** Miriam Abele^1,2^, Etienne Doll^3,4^, Florian P. Bayer^2^, Chen Meng^1^, Klaus Neuhaus^4^, Siegfried Scherer^3^, Bernhard Kuster^1,2^, Christina Ludwig^1 *^

1. Bavarian Center for Biomolecular Mass Spectrometry (BayBioMS), TUM School of Life Sciences, Technical University of Munich, 85354 Freising, Germany
2. Chair of Proteomics and Bioanalytics, TUM School of Life Sciences, Technical University of Munich, 85354 Freising, Germany
3. Research Department Molecular Life Sciences, TUM School of Life Sciences, 85354 Freising, Germany
4. Core Facility Microbiome, ZIEL – Institute for Food & Health, TUM School of Life Sciences, Technical University of Munich, 85354 Freising, Germany

* corresponding author, tina.ludwig@tum.de

Supplementary Figures:

Figure S1

Figure S2

Figure S3

Figure S4

Figure S5

Figure S6

Figure S7

Figure S8

Figure S9


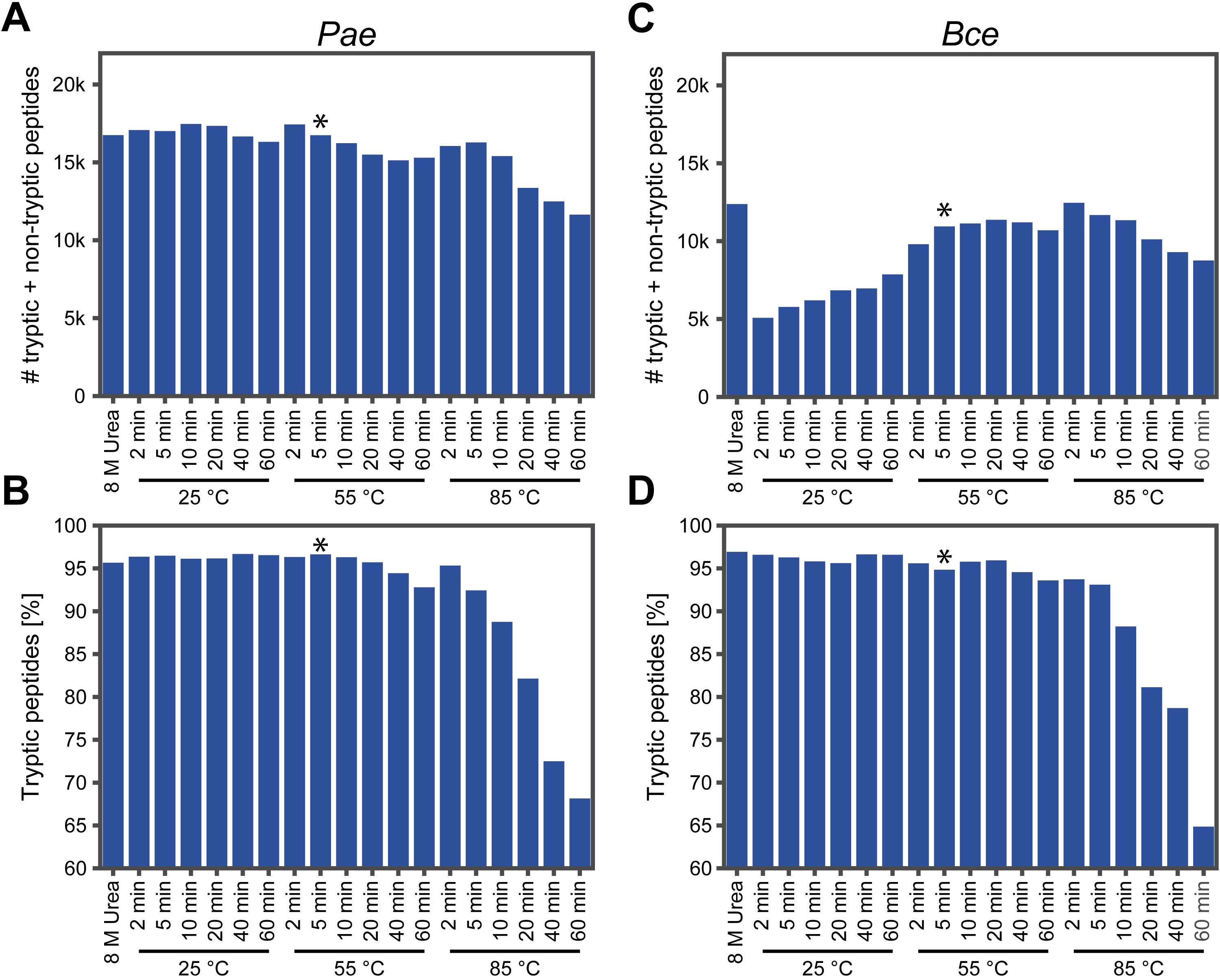


FIG. S1. **Evaluation of TFA-induced hydrolysis of proteins.**  (A) Number of tryptic and non-tryptic peptides from an unspecific MaxQuant search for *Pseudomonas aeruginosa* for various incubation times (2-60 minutes) incubated at various temperatures (25-85°C). (B) Percentage of tryptic peptides compared to all detected peptides (tryptic and non-tryptic). (C, D) Same as (A, B), but for *Bacillus cereus*. Asterisks mark the optimal condition used for all the following experiments carried out in this study.


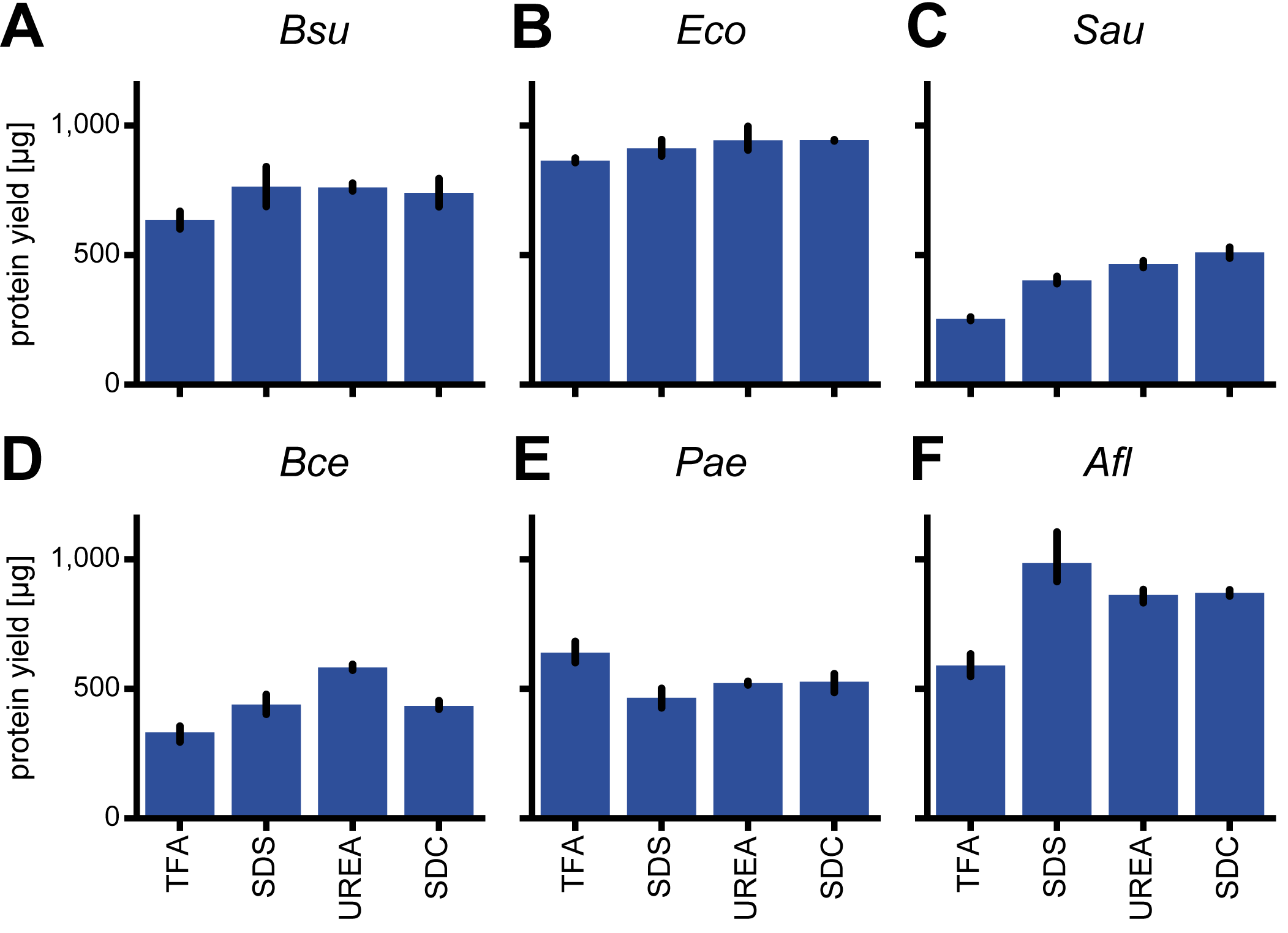


FIG. S2. **Total protein yield for the applied cell lysis strategies (8 M Urea, 2% SDS, 2% SDC, 100% TFA).** Protein yields were determined for the six representative bacterial species *Bacillus subtilis* (A), *Escherichia coli* (B), *Staphylococcus aureus* (C), *Bacillus cereus* (D), *Pseudomonas aeruginosa* (E), and *Anoxybacillus flavithermus* (F).


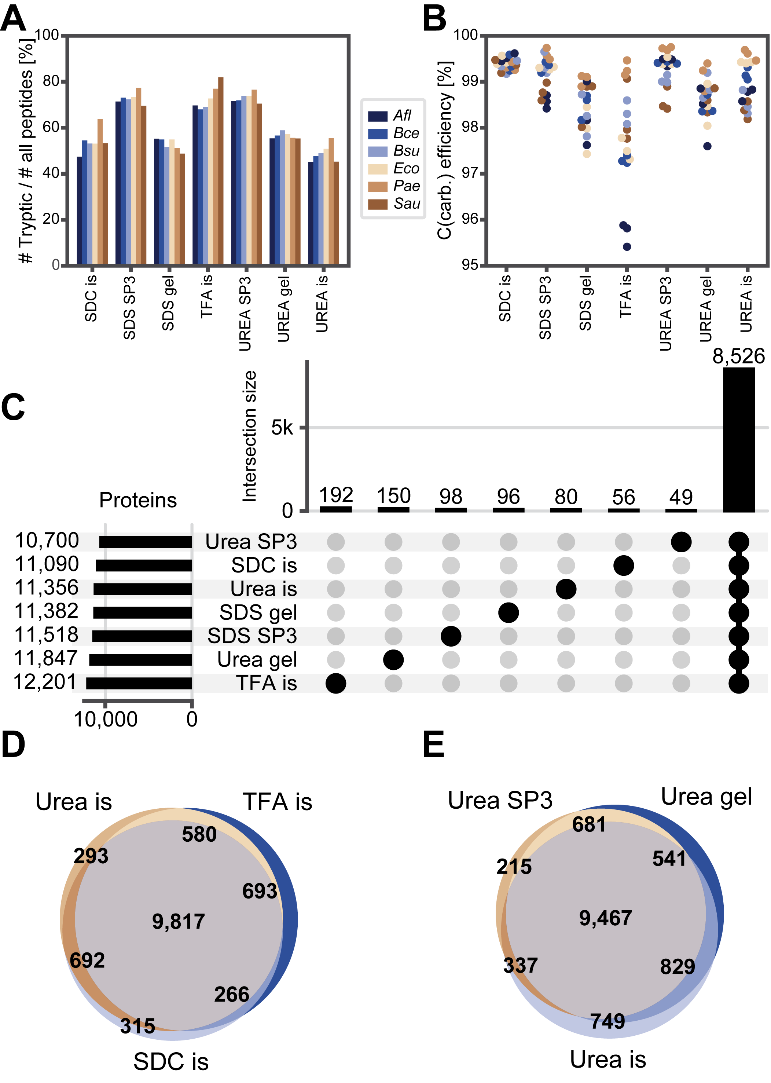


FIG. S3. **Performance evaluation of seven sample preparation workflows.** (A) Percentage of fully tryptic peptides (no missed cleavages) compared to the total number of tryptic peptide identifications (including missed-cleaved peptides). (B) Carbamidomethylation efficiency of the seven tested sample preparation protocols. (C) Upset plot depicting the proteins from all six species that were detected in all seven tested protocols, as well as proteins that were detected exclusively in one protocol. (D) Venn diagram showing the overlap of proteins from all six species in the three tested in-solution digestion protocols. (E) Venn diagram showing the overlap of proteins from all six species for samples lysed with 8 M Urea and digested with varying protocols.


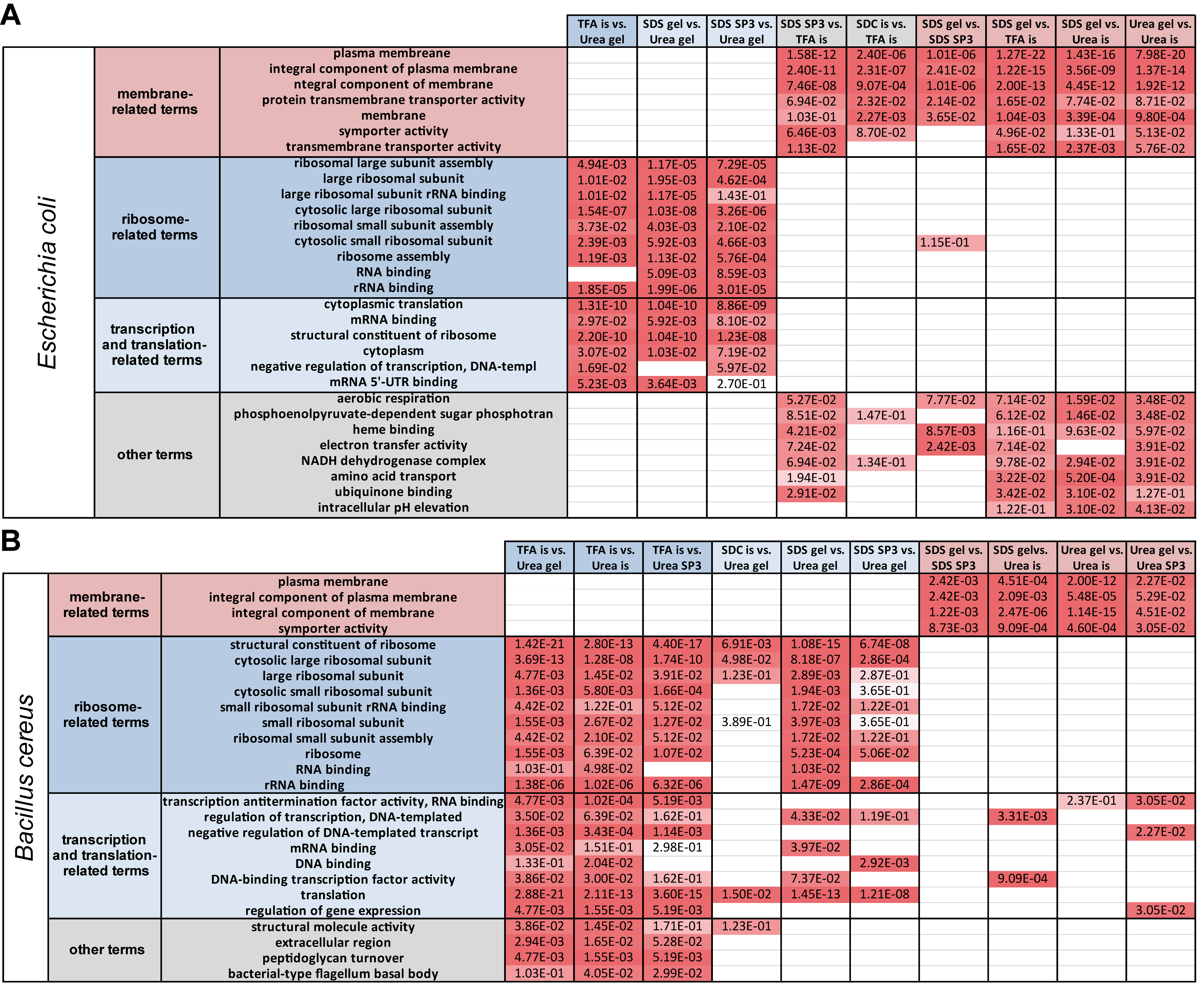


FIG. S4. **Gene ontology terms enriched for the seven tested sample preparation protocols.** (A) Gene ontology terms for proteins significantly upregulated (log_10_ fold change > 2, adjusted p-value < 0.05) in *Escherichia coli*. Matrix was filtered for > 4 significant hits detected in minimum two sample preparation comparisons per GO term. White space indicates no enrichment of this term. (B) Same as (A), but for *Bacillus cereus*.


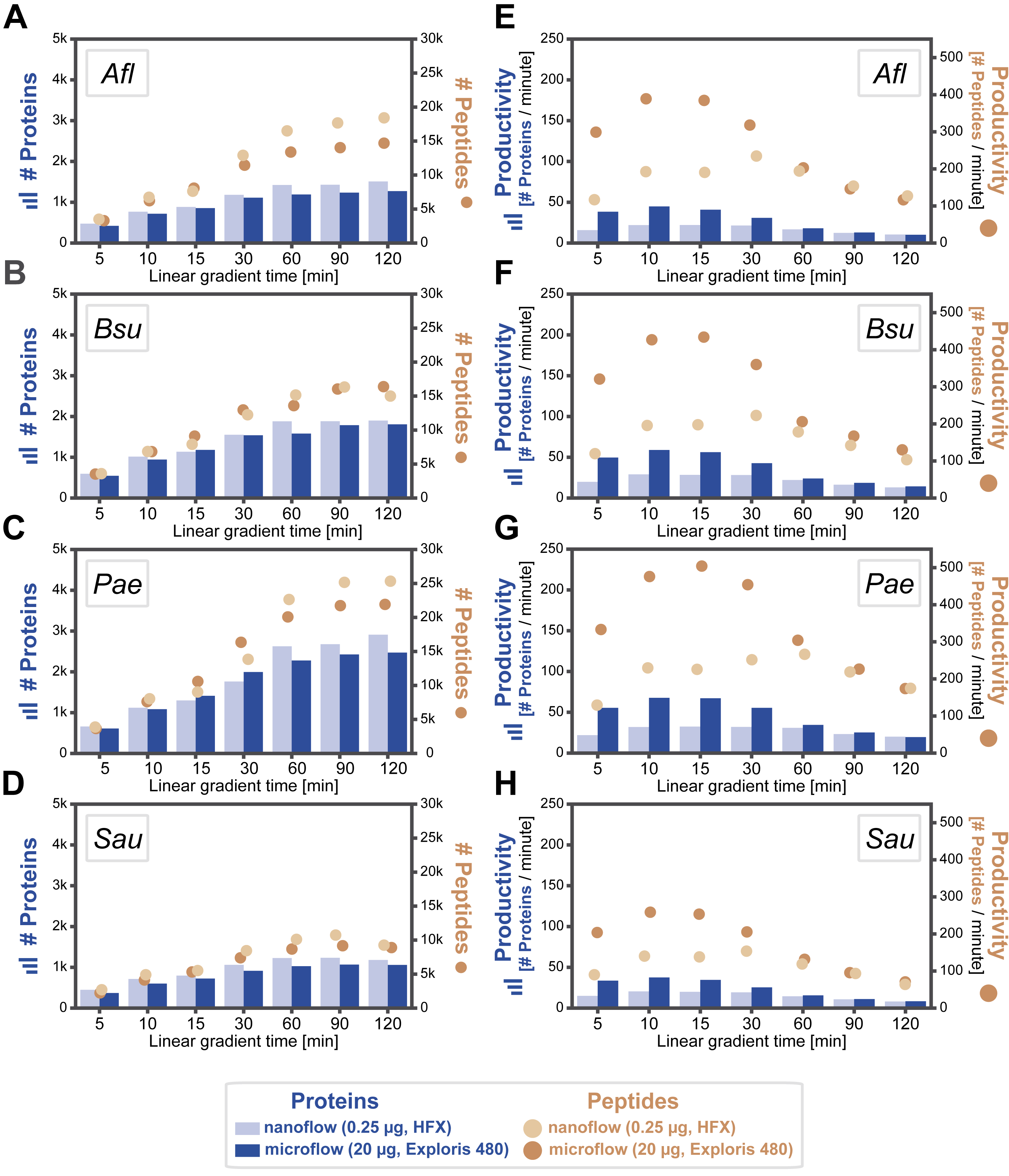


FIG. S5. **Comparison of micro- and nanoflow LC-MS/MS.** (A-D) Protein (bars) and peptide (circles) identifications for various linear LC gradients with nano flowrates (300 nl/min, light) and micro flowrates (50 µl/min, dark) acquired in data-dependent acquisition mode. (E-H) Productivity of protein and peptide identification per minute as a function of total method time (linear gradient time plus overhead time). The x-axis label denotes the linear gradient time only.


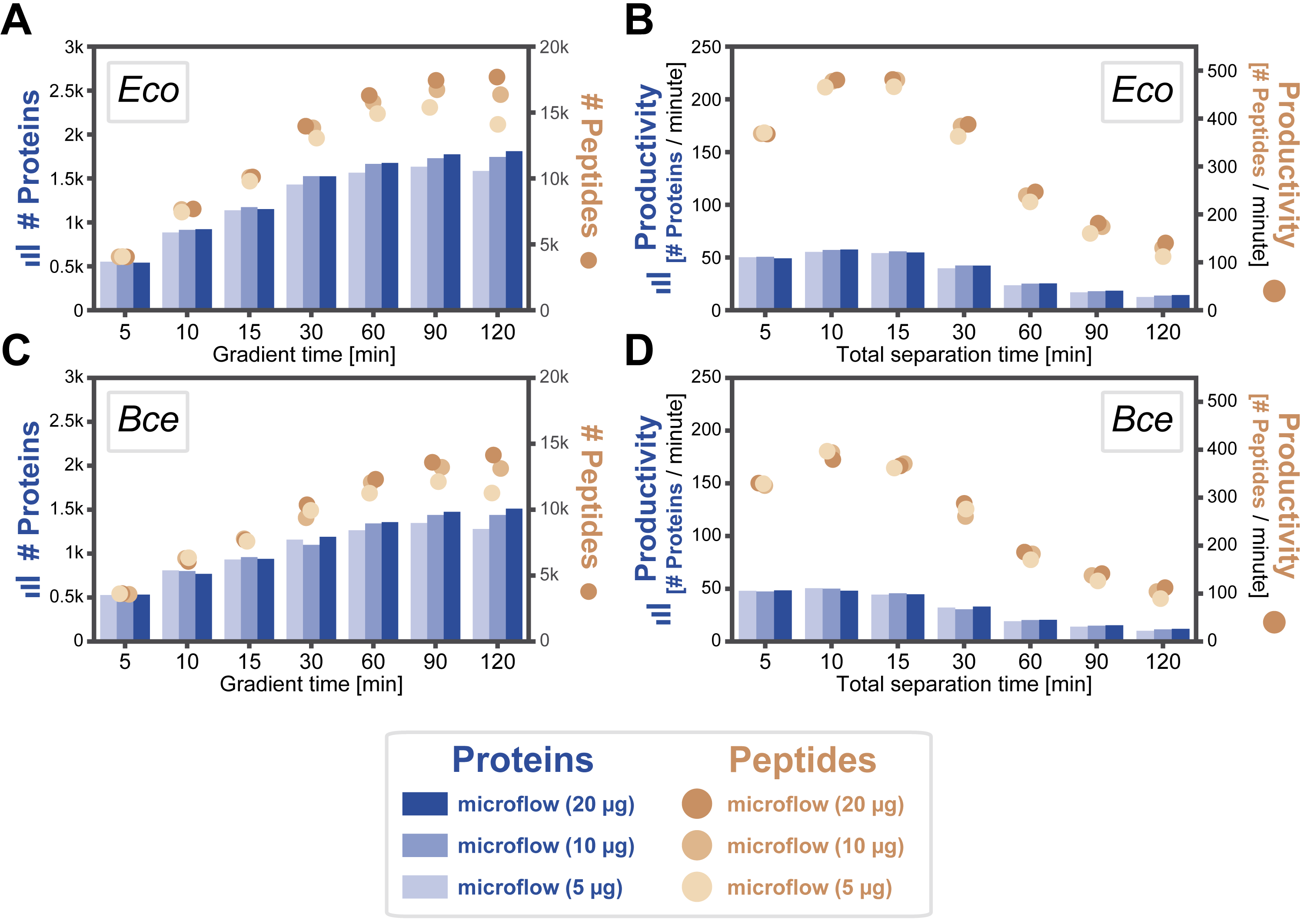


FIG. S6. **Influence of peptide injection amounts on protein and peptide identifications and identification rates.** (A-B) The number of proteins (bars, left axis) and peptides (circles, right axis) identified for *Escherichia coli* as a function of gradient time for various injection amounts. (B) Productivity of protein and peptide identification per minute as a function of total method time (linear gradient time plus overhead time). The x-axis label denotes the linear gradient time only. (C-D) Same as panels (A, B), but for *Bacillus cereus*.


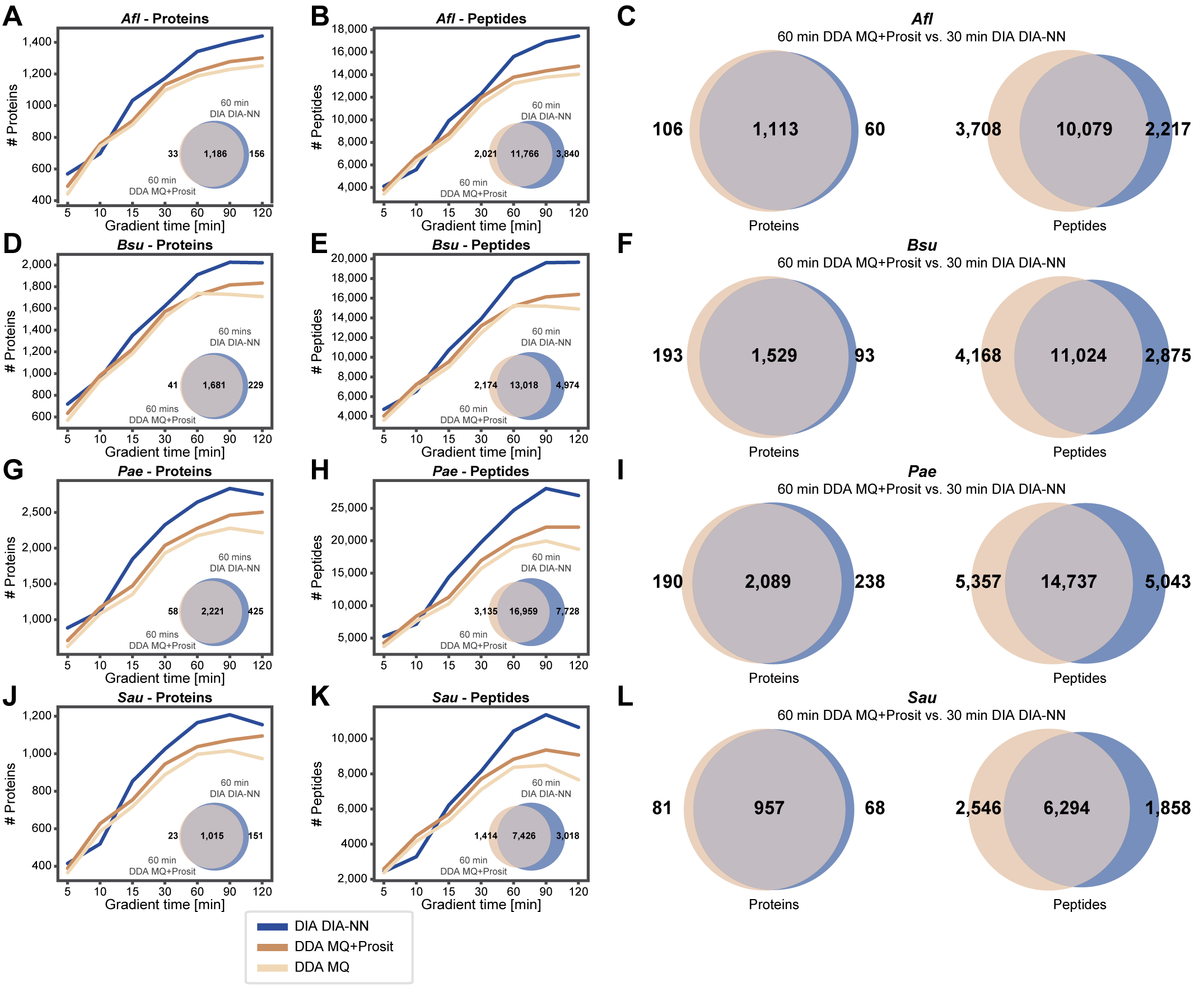


FIG. S7. **Performance evaluation of data-dependent and data-independent acquisition.** (A) Number of protein identifications for *Anoxybacillus flavithermus*. DIA data were searched with an *in silico* predicted library by DIA-NN (DIA DIA-NN, dark blue line). Ten micrograms of peptides were injected. MaxQuant searched DDA data at 1% protein-level FDR (DDA MQ, brown line). Additionally, DDA data was searched by MaxQuant followed by a Prosit re-scoring step (DDA MQ+Prosit, beige line). Venn diagrams represent the protein overlap in a 60-minute DIA DIA-NN experiment versus a 60-minute DDA MQ+Prosit experiment. (B) Same as (A), but for peptide identifications. (C) Venn diagrams show the overlap between a 60-minute DDA MQ+Prosit method and a 30-minute DIA DIA-NN method. (D, E, F) Same as panels (A, B, C), but for *Bacillus subtilis,* (G, H, I) for *Pseudomonas aeruginosa*, (J, K, L) for *Staphylococcus aureus*.


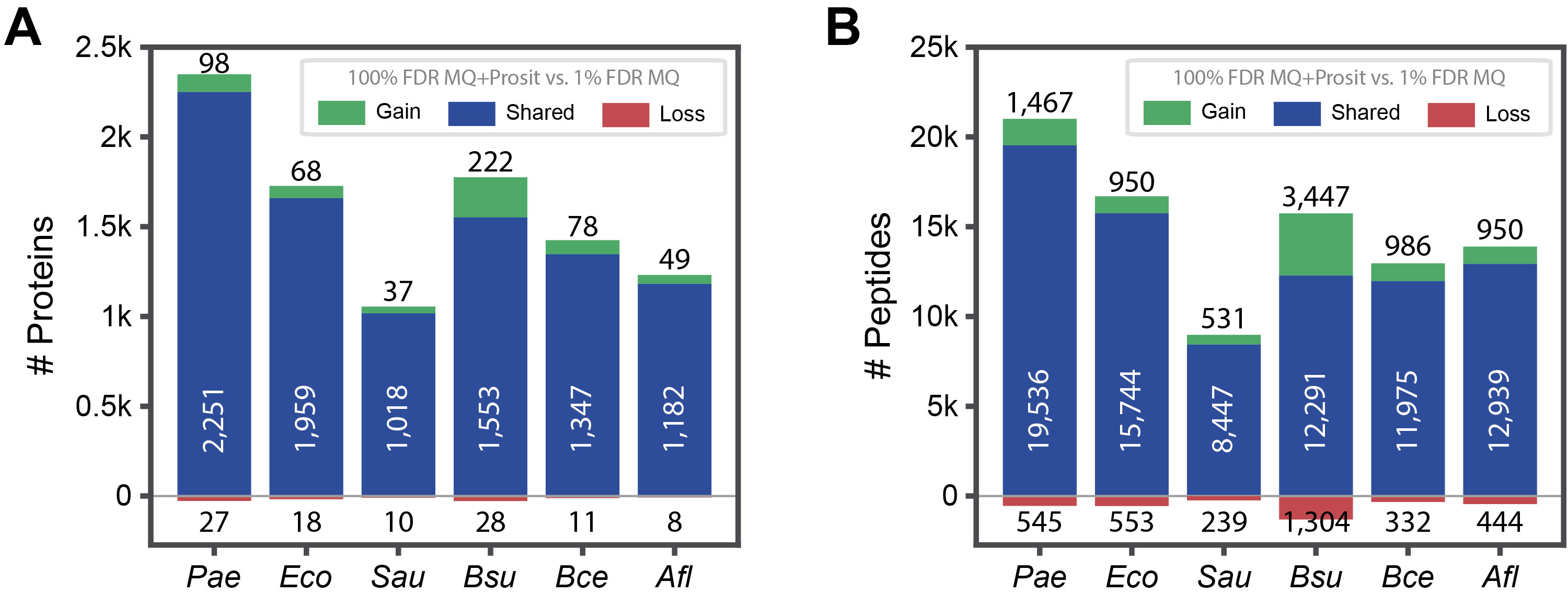


FIG. S8. **Performance evaluation of Prosit rescoring.** (A) Bar-type Venn diagram comparing protein identifications in a 60-minute microflow LC gradient for a 1% protein-level FDR MaxQuant (MQ) search versus a 100% protein-level FDR MQ search with Prosit re-scoring and a picked-group FDR estimation approach (1% FDR). Proteins exclusively identified in 100% FDR MQ+Prosit are highlighted in green, shared in blue, and proteins exclusively identified in the 1% FDR MQ search in red. (B) Same as (A), but for peptide identifications. *Pae*: *Pseudomonas aeruginosa*, *Eco*: *Escherichia coli*, *Sau*: *Staphylococcus aureus*, *Bsu*: *Bacillus subtilis*, *Bce*: *Bacillus cereus*, *Afl*: *Anoxybacillus flavithermus*.


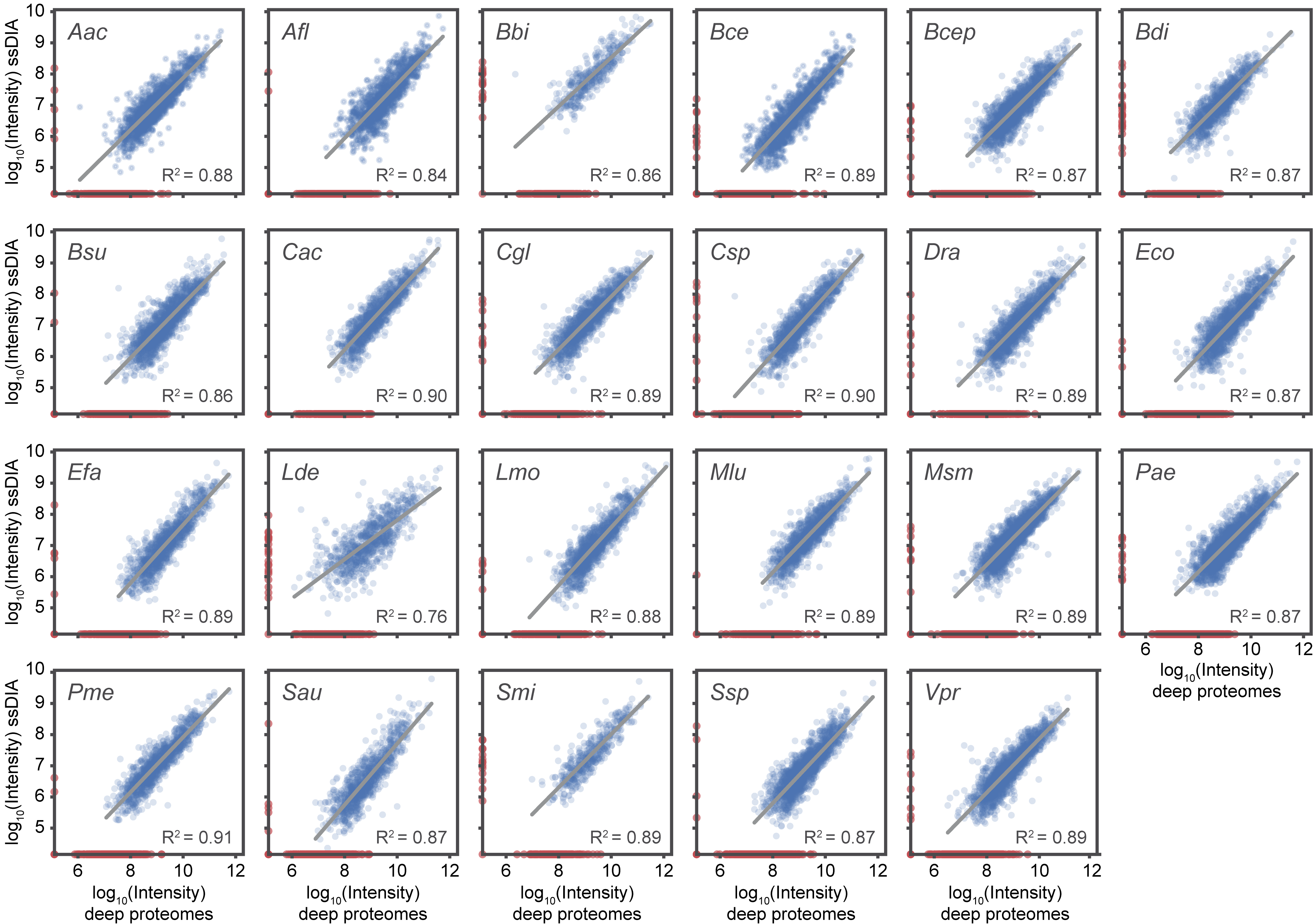


FIG. S9 **Qualitative and quantitative comparison of deep proteomes versus single-shot DIA.** Scatter plot of protein intensities (log_10_ transformed) detected by deep proteome profiling (peptide fractionation, 6 h measurement time, nanoflow, DDA-MS/MS) versus 30-minute single shot DIA measurements (no fractionation, 30 min measurement time, microflow, DIA-MS/MS). The correlation coefficient (R^2^) of proteins quantified in both samples (blue dots) is shown in each panel’s lower, right corner. Red-coloured proteins were found in only one of the two experiments.
