# Supplementary material for: Unified Workflow for the Rapid and In-Depth Characterization of Bacterial Proteomes
Source: Mol Cell Proteomics. 2023 Jun 29;22(8):100612. doi: 10.1016/j.mcpro.2023.100612 (PMC10407251; doi:10.1016/j.mcpro.2023.100612)
Supplement: Supplemental Tables [file mmc2.docx]

**Unified workflow for the rapid and in-depth characterization of bacterial proteomes** Miriam Abele^1,2^, Etienne Doll^3,4^, Florian P. Bayer^2^, Chen Meng^1^, Nina Lomp^1^, Klaus Neuhaus^4^, Siegfried Scherer^3^, Bernhard Kuster^1,2^, Christina Ludwig^1 *^

1. Bavarian Center for Biomolecular Mass Spectrometry (BayBioMS), TUM School of Life Sciences, Technical University of Munich, 85354 Freising, Germany
2. Chair of Proteomics and Bioanalytics, TUM School of Life Sciences, Technical University of Munich, 85354 Freising, Germany
3. Research Department Molecular Life Sciences, TUM School of Life Sciences, 85354 Freising, Germany
4. Core Facility Microbiome, ZIEL – Institute for Food & Health, TUM School of Life Sciences, Technical University of Munich, 85354 Freising, Germany

* corresponding author, tina.ludwig@tum.de

Supplementary Tables:

Supplementary Table S1

Supplementary Table S2

Supplementary Table S3

Supplementary Table S4

Supplementary Table S5

Supplementary Table S6

Supplementary Table S7

Supplementary Table S8

Supplementary Table S9

Supplementary Table S10

| ***Supplementary Table S1***  *Detailed parameters for DIA and DDA methods.* | | | | | | | |
| --- | --- | --- | --- | --- | --- | --- | --- |
| **Method** | **Mass-spectrometer** | **LC flow rate [µg/min]** | **Time [min]** | **Buffer B [%]** | **Top N or cycle time** | **Dynamic exclusion [sec]** | **# windows** |
| 5 min DDA | Q Exactive HFX | 0.3 | 10 - 11 - 16 - 17 | 2 - 4 - 32 - 80 | N = 18 | 10 | - |
| 10 min DDA | Q Exactive HFX | 0.3 | 10 – 11 - 21 - 23 | 2 - 4 - 32 - 80 | N = 18 | 10 | - |
| 15 min DDA | Q Exactive HFX | 0.3 | 10 – 11 - 26 - 27 | 2 - 4 - 32 - 80 | N = 18 | 10 | - |
| 30 min DDA | Q Exactive HFX | 0.3 | 10 - 11 - 41 - 43 | 2 - 4 - 32 - 80 | N = 18 | 10 | - |
| 60 min DDA | Q Exactive HFX | 0.3 | 10 - 11 - 71 - 73 | 2 - 4 - 32 - 80 | N = 18 | 25 | - |
| 90 min DDA | Q Exactive HFX | 0.3 | 10.0 - 11.0 - 101.0 - 103.0 | 2 - 4 - 32 - 80 | N = 18 | 10 | - |
| 120 min DDA | Q Exactive HFX | 0.3 | 10 - 11 - 131 - 133 | 2 - 4 - 32 - 80 | N = 18 | 30 | - |
| 5 min DDA | Exploris 480 | 50 | 0.19 - 0.2 - 5.2 - 5.4 | 1 - 3 - 28 - 90 | 1 sec | 30 | - |
| 10 min DDA | Exploris 480 | 50 | 0.19 - 0.2 - 10.2 - 10.4 | 1 - 3 - 28 - 90 | 1 sec | 30 | - |
| 15 min DDA | Exploris 480 | 50 | 0.19 - 0.2 - 15.2 - 15.4 | 1 - 3 - 28 - 90 | 1 sec | 30 | - |
| 30 min DDA | Exploris 480 | 50 | 0.19 - 0.2 - 30.2 - 30.4 | 1 - 3 - 28 - 90 | 1 sec | 30 | - |
| 60 min DDA | Exploris 480 | 50 | 0.19 - 0.2 - 50.2 - 60.2 - 60.4 | 1 - 3 - 24 - 31 - 90 | 1.2 sec | 30 | - |
| 90 min DDA | Exploris 480 | 50 | 0.19 - 0.2 - 70.2 - 90.2 - 60.4 | 1 - 3 - 20 - 31 - 90 | 1.5 sec | 35 | - |
| 120 min DDA | Exploris 480 | 50 | 0.19 - 0.2 - 90.2 - 120.2 - 60.4 | 1 - 3 - 17 - 31 - 90 | 1.6 sec | 40 | - |
| 5 min DIA | Exploris 480 | 50 | 0.19 - 0.2 - 5.2 - 5.4 | 1 - 3 - 28 - 90 | - | - | 10 |
|  |  |  |  |  |  |  |  |
| 10 min DIA | Exploris 480 | 50 | 0.19 - 0.2 - 10.2 - 10.4 | 1 - 3 - 28 - 90 | - | - | 10 |
| 15 min DIA | Exploris 480 | 50 | 0.19 - 0.2 - 15.2 - 15.4 | 1 - 3 - 28 - 90 | - | - | 20 |
| 30 min DIA | Exploris 480 | 50 | 0.19 - 0.2 - 30.2 - 30.4 | 1 - 3 - 28 - 90 | - | - | 25 |
| 60 min DIA | Exploris 480 | 50 | 0.19 - 0.2 - 50.2 - 60.2 - 60.4 | 1 - 3 - 24 - 31 - 90 | - | - | 40 |
| 90 min DIA | Exploris 480 | 50 | 0.19 - 0.2 - 70.2 - 90.2 - 60.4 | 1 - 3 - 20 - 31 - 90 | - | - | 50 |
| 120 min DIA | Exploris 480 | 50 | 0.19 - 0.2 - 90.2 - 120.2 - 60.4 | 1 - 3 - 17 - 31 - 90 | - | - | 50 |

| ***Supplementary Table S2***  *Overview of all sample preparation protocols and their methodological differences.* | | | | | | |
| --- | --- | --- | --- | --- | --- | --- |
| **Protocol** | **Lysis buffer** | **Beads beating** | **Heat** | **Workflow** | **Reduction reagent** | **Use of a pipetting robot** |
| **SDS gel** | 2% SDS | + / - * | 10 min  at 95 °C | gel | DTT | - |
| **SDS SP3** | 2% SDS | + / - * | 10 min  at 95 °C | SP3 | TCEP | + |
| **Urea is** | 8 M Urea | + / - * | 10 min  at 95 °C | Is | DTT | - |
| **Urea gel** | 8 M Urea | + / - * | 10 min  at 95 °C | gel | DTT | - |
| **Urea SP3** | 8 M Urea | + / - * | 10 min  at 95 °C | SP3 | TCEP | + |
| **SDC is** | 2% SDC | + / - * | 10 min  at 95 °C | Is | DTT | - |
| **TFA is** | 100% TFA | + / - * | 10 min  at 95 °C | is | TCEP | - |
| *SDS = Sodium dodecyl sulfate, SDC = Sodium deoxycholate, TFA = trifluoroacetic acid, gel = in gel digest, SP3 = single-pot, solid-phase-enhanced sample preparation, is = in solution digest* | | | | | | |

* Samples with beads beating were used for LC-MS/MS measurement. Samples without beads beating were only used for lysis efficiency testing, because they partly contained surviving cells and/or spores.

| ***Supplementary Table S3***  *Strains and their respective cultivation conditions used during this study. All bacteria were cultivated on agar plates. Note that Escherichia coli strain MG1655 was used for all optimization steps (Fig. 2 - 4) and strain DSM 30083 was used for the diversity set (Fig. 5). Bacillus cereus and Escherichia coli were used for workflow optimization (Fig. 2 - 4) and for comparative proteome analysis (Fig. 6).* | | | |
| --- | --- | --- | --- |
| Bacterium | Abb. | Strain ID | Cultivation condition |
| ***Acetobacter aceti*** | *Aac* | DSM 3508 T | MRS, 30 °C, aerob |
| ***Anoxybacillus flavithermus*** | *Afl* | in house, G10622* | TSA, 55 °C, aerob |
| ***Bacillus cereus (workflow optimization & deep proteome profiling)*** | *Bce* | DSM 31 T, ATCC 14579 | TSA, 30 °C, aerob |
| ***Bacillus cereus (comparative proteome analysis)*** | *Bce* | DSM 31 T, ATCC 14579 | TSA, 30°C, aerob  TSB, 30°C, aerob  BHI, 30 °C, aerob  CB, 30°C, aerob |
| ***Bacillus subtilis*** | *Bsu* | DSM 10 T, ATCC 6051 | TSA, 30 °C, aerob |
| ***Brevundimonas diminuta*** | *Bdi* | DSM 7234 T, ATCC 11568 | TSA, 30 °C, aerob |
| ***Burkholderia cepacia*** | *Bcep* | DSM 7288 T, ATCC 25416 | TSA, 30 °C, aerob |
| ***Clostridium sporogenes*** | *Csp* | DSM 795 T, ATCC 3584 | RCM, 37 °C, anaerob |
| ***Corynebacterium glutamicum*** | *Cgl* | DSM 20300, ATCC 13032 | TSA, 30 °C, aerob |
| ***Cutibacterium acnes*** | *Cac* | DSM 16379 T | CB, 30 °C, anaerob |
| ***Deinococcus radiodurans*** | *Dra* | DSM 20539 T, ATCC 13939 | TSA, 30 °C, aerob |
| ***Enterococcus faecalis*** | *Efa* | DSM 20478 T, ATCC 19433 | TSA, 30 °C, aerob |
| ***Escherichia coli***  ***(workflow optimization)*** | *Eco* | MG1655, DSM **18039** | TSA, 30 °C, aerob |
| ***Escherichia coli***  ***(deep proteome profiling)*** | *Eco* | DSM 30083 T, ATCC 11775 | TSA, 30 °C, aerob |
| ***Escherichia coli***  ***(comparative proteome analysis)*** | *Eco* | DSM 30083 T, ATCC 11775 | TSA, 30°C, aerob  TSB, 30°C, aerob  BHI, 30 °C, aerob  CB, 30°C, aerob |
| ***Lactobacillus delbrueckii ssp. Delbrueckii*** | *Lde* | DSM 20074 T, ATCC 9649 | MRS, 37 °C, microaerophil |
| ***Listeria monocytogenes*** | *Lmo* | DSM 20600 T, ATCC 15313 | TSA, 30 °C, aerob |
| ***Micrococcus luteus*** | *Mlu* | DSM 20030 T, ATCC 4698 | TSA, 30 °C, aerob |
| ***Mycolicibacterium smegmatis*** | *Msm* | DSM 43756 T, NCTC 8159 | TSA, 30 °C, aerob |
| ***Prevotella melaninogenica*** | *Pme* | DSM 7089 T, ATCC 25845 | CB, 30 °C, anaerob |
| ***Pseudomonas aeruginosa*** | *Pae* | DSM 19880, PA01** | TSA, 30 °C, aerob |
| ***Sphingobacterium spiritivorum*** | *Ssp* | DSM 11722 T, ATCC 33861 | TSA, 30 °C, aerob |
| ***Staphylococcus aureus*** | *Sau* | DSM 20231 T, ATCC12600 | TSA, 30 °C, aerob |
| ***Streptococcus mitis*** | *Smi* | DSM 12643 T, ATCC 49456 | TSA. 37 °C, microaerophil |
| ***Vibrio proteolyticus*** | *Vpr* | DSM 30189, ATCC 15338 | TSA, 30 °C, aerob |
| ***Bifidobacteirum bifidum*** | *Bbi* | DSM 20456 T, ATCC 29521 | BHI, 37 °C, anaerob |
| *Abb. = Abbreviation, T = Type strain, MRS = De Man* MRS De Man, Rogosa and Sharpe agar, TSA = tryptic say agar, RCM = Reinforced Clostridial Medium, BHI = Brain Heart Infustion agar, CB = Columbia Blood agar, TSB = Tryptic Soy Broth, * Dettling et al., 2020, ** the strain is the same strain (duplicate) as DSM 22655 (more information on the DSMZ homepage. | | | |

| ***Supplementary Table S4***  *Mass list table for DIA methods on an Orbitrap Exploris 480 (ThermoFisher, Tune application 3.1.279.9).* | | | | |
| --- | --- | --- | --- | --- |
| Method | Number of windows | Isolation window center [m/z] | Charge z | Isolation window size [m/z] |
| **DIA 5 minutes** | 10 | 391.5  441.5  479.0  515.5  553.0  593.0  639.5  698.0  780.5  1065.0 | 2  2  2  2  2  2  2  2  2  2 | 63  39  38  37  40  42  53  66  101  470 |
| **DIA 10 minutes** | 10 | 391.5  441.5  479.0  515.5  553.0  593.0  639.5  698.0  780.5  1065.0 | 2  2  2  2  2  2  2  2  2  2 | 63  39  38  37  40  42  53  66  101  470 |
| **DIA 15 minutes** | 20 | 381.0  412.0  432.5  451.5  470.0  488.5  507.0  525.0  543.5  563.0  582.5  603.0  625.5  651.5  680.0  712.5  752.5  802.5  877.0  1111.5 | 2  2  2  2  2  2  2  2  2  2  2  2  2  2  2  2  2  2  2  2 | 42  22  21  19  20  19  20  18  21  20  21  22  25  29  30  37  45  57  94  377 |
| **DIA 30 minutes** | 25 | 378.0  405.5  423.0  438.0  453.0  468.0  483.0  498.0  512.5  526.5  541.5  557.0  572.5  588.5  605.0  623.0  643.0  666.0  689.0  715.5  747.5  786.0  834.0  907.5  1127.0 | 2  2  2  2  2  2  2  2  2  2  2  2  2  2  2  2  2  2  2  2  2  2  2  2  2 | 36  21  16  16  16  16  16  16  15  15  17  16  17  17  18  20  23  24  24  31  35  44  54  95  346 |
| **DIA 60 minutes** | 40 | 373.0  393.5  407.0  417.5  427.5  437.5  447.0  456.0  465.0  474.5  484.0  493.0  502.5  512.0  520.5  529.0  538.5  548.5  558.0  567.5  577.5  587.5  597.5  608.0  619.5  631.5  644.5  658.5  672.5  687.0  703.0  721.0  740.5  762.5  788.5  816.5  849.5  896.0  967.0  1155.0 | 2  2  2  2  2  2  2  2  2  2  2  2  2  2  2  2  2  2  2  2  2  2  2  2  2  2  2  2  2  2  2  2  2  2  2  2  2  2  2  2 | 26  17  12  11  11  11  10  10  10  11  10  10  11  10  9  10  11  11  10  11  11  11  11  12  13  13  15  15  15  16  18  20  21  25  29  29  39  56  88  290 |
| **DIA 90 minutes** | 50 | 371.0  388.5  401.0  411.0  419.0  426.5  434.5  442.0  449.0  456.5  464.0  471.5  479.0  486.5  4494.0  501.5  509.0  516.0  522.5  529.5  537.5  545.5  553.5  561.0  568.5  576.5  584.5  592.5  600.5  609  618.5  628.0  638.0  649.0  660.0  671.5  683.5  695.0  707.5  722.5  738.5  755.5  774.5  796.0  819.0  845.5  880.0  927.0  994.5  1167.0 | 2  2  2  2  2  2  2  2  2  2  2  2  2  2  2  2  2  2  2  2  2  2  2  2  2  2  2  2  2  2  2  2  2  2  2  2  2  2  2  2  2  2  2  2  2  2  2  2  2  2 | 22  15  12  10  8  9  9  8  8  9  8  9  8  9  8  9  8  8  7  9  9  9  9  8  9  9  9  9  9  10  11  10  12  12  12  13  13  12  15  17  17  19  21  24  24  31  40  56  81  266 |
| **DIA 120 minutes** | 50 | 371.0  388.5  401.0  411.0  419.0  426.5  434.5  442.0  449.0  456.5  464.0  471.5  479.0  486.5  4494.0  501.5  509.0  516.0  522.5  529.5  537.5  545.5  553.5  561.0  568.5  576.5  584.5  592.5  600.5  609  618.5  628.0  638.0  649.0  660.0  671.5  683.5  695.0  707.5  722.5  738.5  755.5  774.5  796.0  819.0  845.5  880.0  927.0  994.5  1167.0 | 2  2  2  2  2  2  2  2  2  2  2  2  2  2  2  2  2  2  2  2  2  2  2  2  2  2  2  2  2  2  2  2  2  2  2  2  2  2  2  2  2  2  2  2  2  2  2  2  2  2 | 22  15  12  10  8  9  9  8  8  9  8  9  8  9  8  9  8  8  7  9  9  9  9  8  9  9  9  9  9  10  11  10  12  12  12  13  13  12  15  17  17  19  21  24  24  31  40  56  81  266 |
|  |  |  |  |  |

| ***Supplementary Table S5***  *Uniprot fasta files for DIA analysis with DIA-NN and DDA analysis with MaxQuant.* | | | | |
| --- | --- | --- | --- | --- |
| **Bacterium** | **Fasta file name** | **Uniprot identifier** | **Download date** | **# of ORFs** |
| ***Acetobacter aceti*** | *acetobacter_aceti_DSM3508.fasta* | UP000032677 | 20221026 | 3,146 |
| ***Anoxybacillus flavithermus*** | *anoxybacillus_flavithermus_G10622.fasta* | in house* | - | 2,747 |
| ***Bacillus cereus*** | *bacillus_cereus_DSM31.fasta* | UP000001417 | 20220329 | 5,240 |
| ***Bacillus subtilis*** | *bacillus_subtilis_DSM10.fasta* | UP000502638 | 20221026 | 4,236 |
| ***Brevundimonas diminuta*** | *brevundimonas_diminuta_DSM7234.fasta* | UP000255513 | 20221026 | 3,305 |
| ***Burkholderia cepacia*** | *burkholderia_cepacia_ATCC25416.fasta* | UP000068183 | 20221026 | 7,446 |
| ***Clostridium sporogenes*** | *clostridium_sporogenes_DSM795.fasta* | UP000486601 | 20221026 | 3,931 |
| ***Corynebacterium glutamicum*** | *corynebacterium_glutamicum_DSM20300.fasta* | UP000000582 | 20220329 | 3,097 |
| ***Cutibacterium acnes*** | *cutibacteirum_acnes_DSM16379.fasta* | UP000000603 | 20220329 | 2,294 |
| ***Deinococcus radiodurans*** | *deinococcus_radiodurans_reference_DSM20539.fasta* | UP000002524 | 20220329 | 3,085 |
| ***Enterococcus faecalis*** | *enterococcus_faecalis_ATCC 19433.fasta* | UP000005586 | 20221026 | 2,967 |
| ***Escherichia coli*** | *escherichia_coli_DSM 30083.fasta; escherichia_coli_MG1655.fasta* | UP000478303  UP000000625 | 20221026  20220523 | 5,098  4,448 |
| ***Lactobacillus delbreuckii ssp. Delbrueckii*** | *lactococcus_delbrueckii_DSM20074.fasta* | UP000051434 | 20220329 | 1,799 |
| ***Listeria monocytogenes*** | *listeria_monocytogenes.fasta* | UP000000817 | 20221026 | 2,844 |
| ***Micrococcus luteus*** | *micrococcus_luteus_DSM20030.fasta* | UP000000738 | 20220329 | 2,207 |
| ***Mycolicibacterium smegmatis*** | *mycolicibacterium_smegmatis_NCTC8159.fasta* | UP000255288 | 20221026 | 6,579 |
| ***Prevotella melaninogenica*** | *prevotella_melaninogenica_DSM7089.fasta* | UP000001498 | 20220329 | 2,288 |
| ***Pseudomonas aeruginosa*** | *pseudomonas_aeruginosa_DSM22644.fasta* | UP000002438 | 20220329 | 5,564 |
| ***Sphingobacterium spiritovorum*** | *sphingobacterium_spiritovorum_ATCC33861.fasta* | UP000006258 | 20220330 | 4,471 |
| ***Staphylococcus aureus*** | *staphylococcus_aueus_DSM20231.fasta* | UP000006386 | 20221026 | 2,583 |
| ***Streptococcus mitis*** | *streptococcus_mitis_NCTC12261.fasta* | UP000016570 | 20221026 | 2,583 |
| ***Vibrio proteolyticus*** | *vibrio_proteolyticus_NBRC13287.fasta* | UP000016570 | 20221026 | 1,694 |
| ***Bifidobacteirum bifidum*** | *bifidobacterium_bifidum_ATCC29521.fasta* | UP000029005 | 20221026 | 1,704 |

| ***Supplementary Table S6***  *Time calculation for 96 samples for the seven tested sample preparation protocols.* | | | | | |
| --- | --- | --- | --- | --- | --- |
| **Sample preparation protocol** | **Lysis**  **[min]** | **Protein concentration [min]** | **Digest [min]** | **Desalting [min]** | **Total time per 96 samples**  **[min]** |
| ***SDS-gel*** | 240 | 180 | 1655 | 120 | 2195 |
| ***SDS-SP3*** | 240 | 180 | 1015 | 270 | 1705 |
| ***Urea-is*** | 250 | 180 | 1020 | 270 | 1720 |
| ***Urea-gel*** | 250 | 180 | 1655 | 120 | 2205 |
| ***Urea-SP3*** | 250 | 180 | 1015 | 210 | 1715 |
| ***SDC-is*** | 240 | 180 | 1020 | 270 | 1710 |
| ***TFA-is*** | 150 | 160 | 925 | 270 | 1505 |

| ***Supplementary Table S7***  *Cost calculation for 96 samples for the seven tested sample preparation protocols.* | | | | | |
| --- | --- | --- | --- | --- | --- |
| **Sample preparation protocol** | **Lysis**  **[€]** | **Protein concentration [€]** | **Digest**  **[€]** | **Desalting**  **[€]** | **Total costs per 96 samples**  **[€]** |
| ***SDS gel*** | 345 | 50 | 340 | - | 735 |
| ***SDS SP3*** | 345 | 50 | 60 | 195 | 650 |
| ***Urea is*** | 345 | 50 | 40 | 190 | 625 |
| ***Urea gel*** | 345 | 50 | 340 | - | 735 |
| ***Urea SP3*** | 345 | 50 | 60 | 195 | 650 |
| ***SDC is*** | 345 | 50 | 40 | 190 | 625 |
| ***TFA is*** | 95 | 30 | 40 | 190 | 345 |

| ***Supplementary Table S8***  *Lysis efficiencies of 100% TFA for 18 diverse bacterial species. Reported colonies are the mean of two replicates if not otherwise stated.* | | | | | |
| --- | --- | --- | --- | --- | --- |
| **Bacterium** | **Lysis strategy** | **Cfu/ml before lysis** | **Detected cfu after lysis** | **Cfu/ml after lysis***** | **Lysis efficiency [%]** |
| ***Acetobacter aceti* DSM 3508^T^** | 100% TFA | 2.94x10^8^* | 0 | <10 | > 99.9 |
| ***Brevibacterium linens* DSM 20425^T^** | 100% TFA | 8.75x10^8^ | 0 | <10 | > 99.9 |
| ***Brevundimonas diminuta* DSM 7234^T^** | 100% TFA | 1.47x10^9^** | 0 | <10 | > 99.9 |
| ***Burkholderia cepacia* DSM 7288^T^** | 100% TFA | 6.60x10^8^ | 0 | <10 | > 99.9 |
| ***Clostridium sporogenes* DSM 795^T^** | 100% TFA | 7.92x10^7^ | 0 | <10 | > 99.9 |
| ***Corynebacterium glutamicum* DSM 20300** | 100% TFA | 1.39x10^9^ | 0 | <10 | > 99.9 |
| ***Cutibacterium acnes* DSM 16379^T^** | 100% TFA | NA | 0 | <10 | > 99.9 |
| ***Deinococcus radiodurans* DSM 20539^T^** | 100% TFA | 1.80x10^8^ | 0 | <10 | > 99.9 |
| ***Enterococcus faecalis* DSM 20478^T^** | 100% TFA | 2.66x10^9^ | 0 | <10 | > 99.9 |
| ***Lactobacillus delbrueckii* DSM 20074^T^** | 100% TFA | NA | 0 | <10 | > 99.9 |
| ***Listeria monocytogenes* DSM 20600^T^** | 100% TFA | 4.95x10^9^ | 0 | <10 | > 99.9 |
| ***Micrococcus luteus* DSM 20030^T^** | 100% TFA | 1.08x10^9^ | 0 | <10 | > 99.9 |
| ***Mycolicibacterium smegmatis* DSM 43756^T^** | 100% TFA | NA | 0 | <10 | > 99.9 |
| ***Prevotella melaninogenica* DSM 7089^T^** | 100% TFA | NA | 0 | <10 | > 99.9 |
| ***Sphingobacterium spiritovorum* DSM 11722^T^** | 100% TFA | 7.05x10^9^ | 0 | <10 | > 99.9 |
| ***Streptococcus mitis* DSM 12643^T^** | 100% TFA | 1.28x10^9^ | 0 | <10 | > 99.9 |
| ***Vibrio proteolyticus* NBRC 13287** | 100% TFA | 9.16x10^10^ | 0 | <10 | > 99.9 |
| ***Bifidobacterium bifidum* DSM 20456^T^** | 100% TFA | NA | 0 | <10 | > 99.9 |
| ** colonies from only one replicate, ** colonies from only two replicates of one dilution were counted, *** From undiluted lysate, only 100 µl were streaked on an agar plate. If no colony-forming units were detected after incubations, this translates to < 10 colonies in one milliliter of lysate, NA too many colonies were grown, T = Type strain* | | | | | |

| ***Supplementary Table S9***  *Set of diverse bacterial species and their selection criteria.* | | | | | |
| --- | --- | --- | --- | --- | --- |
| Bacterium | abb. | Phylum | Gram staining | Selection criterium | Safety level |
| ***Deinococcus radiodurans*** | *Dra* | Deinococcus-Thermus | G+/G- | Tax | S1 |
| ***Sphingobacterium spiritovorum*** | *Ssp* | Bacteroidetes | G- | Tax/P | S2 |
| ***Prevotella melaninogenica*** | *Pme* | Bacteroidetes | G- | Tax/P | S2 |
| ***Micrococcus luteus*** | *Mlu* | Actinobacteria | G+ | Tax/A/K | S1 |
| ***Bifidobacterium bifidum*** | *Bbi* | Actinobacteria | G+ | Tax/T/A | S1 |
| ***Cutibacterium acnes*** | *Cac* | Actinobacteria | G+ | P | S2 |
| ***Corynebacterium glutamicum*** | *Cgl* | Actinobacteria | G+ | Tax/P/A | S1 |
| ***Mycobacterium smegmatis*** | *Msm* | Actinobacteria | G+ | Tax/P/A | S2 |
| ***Burkholderia cepacia*** | *Bcep* | Proteobacteria | G- | Tax/P | S2 |
| ***Acetobacter aceti*** | *Aac* | Proteobacteria | G- | Tax/T | S1 |
| ***Brevundimonas diminuta*** | *Bdi* | Proteobacteria | G- | Tax | S1 |
| ***Pseudomonas aeruginosa*** | *Pse* | Proteobacteria | G- | Tax/P | S2 |
| ***Escherichia coli*** | *Eco* | Proteobacteria | G- | Tax/P | S2 |
| ***Vibrio proteolyticus*** | *Vpr* | Proteobacteria | G- | Tax/M | S1 |
| ***Clostridium sporogenes*** | *Csp* | *Firmicutes* | G+ | Tax/P | S2 |
| ***Enterococcus faecalis*** | *Efa* | Firmicutes | G+ | Tax/P | S2 |
| ***Lactobacillus delbruecki*** | *Lde* | Firmicutes | G+ | Tax/T | S2 |
| ***Streptococcus mitis*** | *Smi* | Firmicutes | G+ | Tax/P | S2 |
| ***Staphylococcus aureus*** | *Sau* | *Firmicutes* | G+ | Tax/P | S2 |
| ***Listeria monocytognes*** | *Lmo* | *Firmicutes* | G+ | Tax/P | S2 |
| ***Anoxybacillus flavithermus*** | *Afl* | Firmicutes | G+ | Tax/T | S1 |
| ***Bacillus cereus*** | *Bce* | Firmicutes | G+ | Tax/P | S2 |
| ***Bacillus subtilis*** | *Bsu* | Firmicutes | G+ | M | S1 |
| *Tax = phylogenetic relationship, M = academic relevance (model organism), T = technological relevance, P = pathogenicity, A = lysis resistance* | | | |  |  |
